# The Proteasome Governs Fungal Morphogenesis via Functional Connections with Hsp90 and cAMP-Protein Kinase A Signaling

**DOI:** 10.1128/mBio.00290-20

**Published:** 2020-04-21

**Authors:** Saif Hossain, Amanda O. Veri, Leah E. Cowen

**Affiliations:** a Department of Molecular Genetics, University of Toronto, Toronto, Ontario, Canada; University of Texas Health Science Center

**Keywords:** *Candida*, Hsp90, fungi, morphogenesis, proteasome, Ras signaling

## Abstract

Protein homeostasis is critical for proliferation and viability of all organisms. For Candida albicans, protein homeostasis also modulates the transition between yeast and filamentous forms, which is critical for virulence. A key regulator of morphogenesis is the molecular chaperone Hsp90, which mediates proteostasis under physiological and stress conditions. Hsp90 regulates morphogenesis by repressing cyclic AMP-protein kinase A (cAMP-PKA) signaling, such that inhibition of Hsp90 causes filamentation in the absence of an inducing cue. We explored the effect of perturbation of another facet of protein homeostasis and discovered that morphogenesis is also regulated by the proteasome, a large 33-subunit protein complex consisting of a 20S catalytic core and two 19S regulatory particles, which controls degradation of intracellular proteins. We identified a conserved role of the proteasome in morphogenesis as pharmacological inhibition of the proteasome induced filamentation of C. albicans and the related species Candida dubliniensis, Candida tropicalis, Candida krusei, and Candida parapsilosis. For C. albicans, genetic depletion of any of 29 subunits of the 19S or 20S particle induced filamentation. Filaments induced by inhibition of either the proteasome or Hsp90 have shared structural characteristics, such as aberrant nuclear content, and shared genetic dependencies, such as intact cAMP-PKA signaling. Consistent with a functional connection between these facets of protein homeostasis that modulate morphogenesis, we observed that proteasome inhibition results in an accumulation of ubiquitinated proteins that overwhelm Hsp90 function, relieving Hsp90-mediated repression of morphogenesis. Together, our findings provide a mechanism whereby interconnected facets of proteostasis regulate C. albicans morphogenesis.

## Abstract (precis)

Fungi cause life-threatening infections and pose a serious threat to human health as there are very few effective antifungal drugs. Candida albicans is a major human fungal pathogen and cause of morbidity and mortality in immunocompromised individuals. A key trait that enables C. albicans virulence is its ability to transition between yeast and filamentous forms. Understanding the mechanisms regulating this virulence trait can facilitate the development of much-needed, novel therapeutic strategies. A key regulator of morphogenesis is the molecular chaperone Hsp90, which is crucial for proteostasis. Here, we expanded our understanding of how proteostasis regulates fungal morphogenesis and identified the proteasome as a repressor of filamentation in C. albicans and related species. Our work suggests that proteasome inhibition overwhelms Hsp90 function, thereby inducing morphogenesis. This work provides a foundation for understanding the role of the proteasome in fungal virulence and offers potential for targeting the proteasome to disarm fungal pathogens.

## INTRODUCTION

Fungi infect billions of people worldwide and kill at least 1.5 million people annually, on par with tuberculosis and malaria (1). One of the leading human fungal pathogens is Candida albicans, an opportunistic pathogen that is a natural member of the human mucosal microbiota and is capable of causing both superficial and life-threatening systemic infections in immunocompromised people, with mortality rates of ∼40% (2). A key virulence trait of C. albicans is its capacity to transition between yeast and filamentous forms in response to diverse host-relevant cues, including exposure to serum, elevated temperature, nutrient limitation, and amino acid starvation (3–5). These morphological transitions are integral for virulence as the majority of mutants that grow exclusively as yeast or filaments have attenuated virulence in systemic infection models (6–8). Yeast and filaments have distinct roles in infection, whereby yeast enable colonization and dissemination through the bloodstream, and filaments enable tissue invasion and deep-seated infection (8). As an environmentally contingent virulence trait, morphogenesis is tightly regulated by complex cellular signaling (9).

Cellular signaling is profoundly influenced by protein homeostasis under physiological conditions and in response to environmental stress. Key regulators of proteostasis are molecular chaperones, which facilitate the folding of newly synthesized proteins, mediate refolding of misfolded proteins, and target damaged and terminally misfolded proteins for degradation, mainly through the ubiquitin-proteasome system (10). Two essential regulators in the proteostasis network are the heat shock transcription factor Hsf1 and the molecular chaperone Hsp90. Hsf1 regulates protein homeostasis via transcriptional regulation of chaperone genes, such as *HSP90*, *HSP70*, and *HSP104*, under basal conditions and in response to thermal stress (41, 42, 49). Hsp90 mediates proteostasis under physiological and stress conditions by binding to client proteins, which include diverse signal transducers, and regulating their stability, maturation, and activation (10). As a consequence of their roles in proteostasis, Hsf1 and Hsp90 influence diverse facets of C. albicans biology, including stress response, drug resistance, and virulence (11).

In addition to their roles in regulating protein homeostasis, both Hsp90 and Hsf1 modulate C. albicans filamentation. C. albicans Hsp90 regulates morphogenesis via multiple signaling pathways, including the following: a regulatory circuit comprised of the transcription factor Hms1, cyclin Pcl1, and cyclin-dependent kinase Pho85 (12); the mitotic exit network (13); and the cyclic AMP-protein kinase A (cAMP-PKA) pathway (14). Hsp90 exerts a repressive effect on cAMP-PKA signaling, which is integral for regulating filamentation in response to diverse cues (5, 9, 14, 15). As such, compromising Hsp90 function induces filamentation in the absence of other inducing cues (14). Filamentation is also induced under conditions of cellular stress that cause an accumulation of misfolded proteins that overwhelm Hsp90’s functional capacity, such as elevated temperature (14). Hsp90’s role in regulating morphogenesis is broadly conserved as inhibition of Hsp90 induces morphogenesis of many fungal species, including Candida dubliniensis, Candida tropicalis, Candida lusitaniae, and Candida auris (16). Modulation of *HSF1* levels also has a profound impact on C. albicans morphogenesis as both overexpression and depletion of *HSF1* induce filamentation, albeit through distinct mechanisms. Overexpression of *HSF1* causes an expansion of transcriptional targets as Hsf1 levels are no longer limiting, allowing Hsf1 to bind heat shock elements (HSE) in the promoter regions of its basal target genes as well as over 1,000 additional targets. These expanded transcriptional targets upon *HSF1* overexpression include many morphogenetic regulators, such as *BRG1* and *UME6*, whose misregulation induces filamentation. Depletion of *HSF1* induces filamentation by impairing Hsp90 function, likely due to misregulation of molecular chaperones and Hsp90 cochaperones (17). Perturbation of Hsf1 or Hsp90 function has a profound impact on an environmentally contingent morphogenetic program, but the impact of other facets of protein homeostasis on this key virulence trait have yet to be explored.

Fundamental to the maintenance of proteostasis is the proteasome, which is a highly conserved multisubunit protein complex comprising of a core 20S particle flanked by two 19S regulatory caps (18, 19). This complex protease is integral for the degradation of misfolded and damaged proteins in the cell, which are targeted to the proteasome through covalent attachment of ubiquitin by a cascade of ubiquitin-processing enzymes (20). Ubiquitinated substrates are detected by the 19S regulatory particle (RP) of the proteasome, composed of a 9-subunit lid and a 10-subunit base, which deubiquitylates substrates to recycle ubiquitin and mediates unfolding and translocation of proteins into the core particle (CP) for hydrolysis. The 14-subunit 20S CP is a barrel-like structure containing an enclosed cavity for the degradation of proteins by the proteolytic active sites (20, 21). Proteasome-mediated protein degradation is critical for many key cellular processes, including regulation of the cell cycle, signaling, transcription, and translation. Although the proteasome has not been extensively characterized in pathogenic fungi, it has been implicated in production of Cryptococcus neoformans capsule, a polysaccharide coat attached to the cell wall that is important for virulence (22), suggesting that the proteasome may modulate fungal virulence (23). The impact of the proteasome on C. albicans growth and virulence traits remains unexplored.

Here, we established the proteasome as a novel regulator of fungal morphogenesis such that pharmacological inhibition of the proteasome induces filamentation of diverse fungi. We performed systematic genetic analysis of the 33 proteasome subunits and determined that the majority are repressors of morphogenesis and are essential for viability. We found that filaments formed in response to proteasome inhibition have aberrant septum formation and nuclear stability and share structural similarities to those formed in response to Hsp90 inhibition. We established that, similar to Hsp90 inhibition, proteasome inhibition induces morphogenesis via cAMP-PKA signaling independent of Efg1, implicating additional downstream transcriptional regulators in morphogenesis. We also found that proteasome inhibition overwhelms the functional capacity of Hsp90. Our findings support the model that perturbing proteasome function relieves Hsp90-mediated repression of cAMP-PKA signaling to trigger filamentation. This work establishes new insights into the impact of proteasome function on fungal biology and uncovers a connection between protein degradation and regulation of a core morphogenetic program important for virulence of a leading human fungal pathogen.

## RESULTS

### Inhibition of the proteasome induces morphogenesis in C. albicans.

Regulated protein degradation by the ubiquitin-proteasome system is a highly conserved process in eukaryotes that is essential for maintaining proteostasis (24). While the proteasome has been well characterized in the model yeast Saccharomyces cerevisiae (20), little is known about the proteasome machinery or function in C. albicans. To provide a comprehensive analysis of the C. albicans proteasome, we utilized S. cerevisiae annotations for the 33 proteasome subunit genes to predict C. albicans proteasome genes (20) (Table 1). These predictions identified C. albicans homologues for all 33 subunits, confirming annotations in the *Candida* Genome Database (25) and identifying three previously uncharacterized genes, *orf19.1058*, *orf19.*2755, and *orf19.3122.2*, as potential proteasome subunit genes (Table 1). Reciprocal BLASTp analysis identified *orf19.1058*, *orf19.*2755, and *orf19.3122.2* as homologous to S. cerevisiae *RPN13* (*P* < 5e−20), *PRE7* (*P* < 5e−141), and *SEM1* (*P* < 8e−09), respectively.

**TABLE 1:** Proteasome components in C. albicans

Component and gene name in S. cerevisiae^*a*^	C. albicans gene			Essentiality in S. cerevisiae
	*orf19* name	Systematic name	Standard name	
20S core particle				
*PRE1*	*orf19.4025*	C5_05310W_A	*PRE1*	Essential
*PRE2*	*orf19.2233*	C2_06820C_A	*PRE2*	Essential
*PRE3*	*orf19.6991*	C3_05560W_A	*PRE3*	Essential
*PRE4*	*orf19.4230*	C5_02230W_A	*PRE4*	Essential
*PRE5*	*orf19.7178*	C7_04020C_A	*PRE5*	Essential
*PRE6*	*orf19.544.1*	CR_04550W_A	*PRE6*	Essential
*PRE7*	*orf19.2755*	C4_02470C_A	*PRE7*	Essential
*PRE8*	*orf19.7335*	CR_09380W_A	*PRE8*	Essential
*PRE10*	*orf19.6582*	C7_01470C_A	*PRE10*	Essential
*PUP1*	*orf19.7605*	CR_10300W_A	*PUP1*	Essential
*PUP2*	*orf19.709*	CR_06750C_A	*PUP2*	Essential
*PUP3*	*orf19.1336*	C7_03390C_A	*PUP3*	Essential
*SCL1*	*orf19.5378*	C3_00770C_A	*SCL1*	Essential
*PRE9*	*orf19.350*	C3_03520C_A	*PRE9*	Nonessential
19S regulatory particle lid				
*RPN3*	*orf19.3054*	C1_03520W_A	*RPN3*	Essential
*RPN5*	*orf19.4032*	C5_05380W_A	*RPN5*	Essential
*RPN6*	*orf19.1299*	C4_03790W_A	*RPN6*	Essential
*RPN7*	*orf19.7286*	CR_08910C_A	*RPN7*	Essential
*RPN8*	*orf19.3168*	C5_02030W_A	*RPN8*	Essential
*RPN11*	*orf19.7264*	C1_14460W_A	*RPN11*	Essential
*RPN12*	*orf19.213*	C2_08930W_A	*RPN12*	Essential
*RPN9*	*orf19.1993*	C2_01320W_A	*RPN9*	Nonessential
*SEM1*	*orf19.3122.2*	C4_06880C_A	*SEM1*	Nonessential
19S regulatory particle base				
*RPT1*	*orf19.441*	C1_05240C_A	*RPT1*	Essential
*RPT2*	*orf19.5440*	C3_00290W_A	*RPT2*	Essential
*RPT3*	*orf19.5793*	C2_03060W_A	*PR26/RPT3*	Essential
*RPT4*	*orf19.482*	CR_04000W_A	*RPT4*	Essential
*RPT5*	*orf19.3123*	C4_06870W_A	*RPT5*	Essential
*RPT6*	*orf19.3593*	C2_08780W_A	*RPT6*	Essential
*RPN1*	*orf19.4956*	C1_13300C_A	*RPN1*	Essential
*RPN2*	*orf19.5260*	C1_12050W_A	*RPN2*	Essential
*RPN10*	*orf19.4102*	C2_06150C_A	*RPN10*	Nonessential
*RPN13*	*orf19.1058*	C1_04230W_A	*RPN13*	Nonessential

a S. cerevisiae annotations were obtained from Finley et al. (20). *PRE2*, *PRE3*, and *PUP1* are proteolytically active in S. cerevisiae.

**FIG 1:**
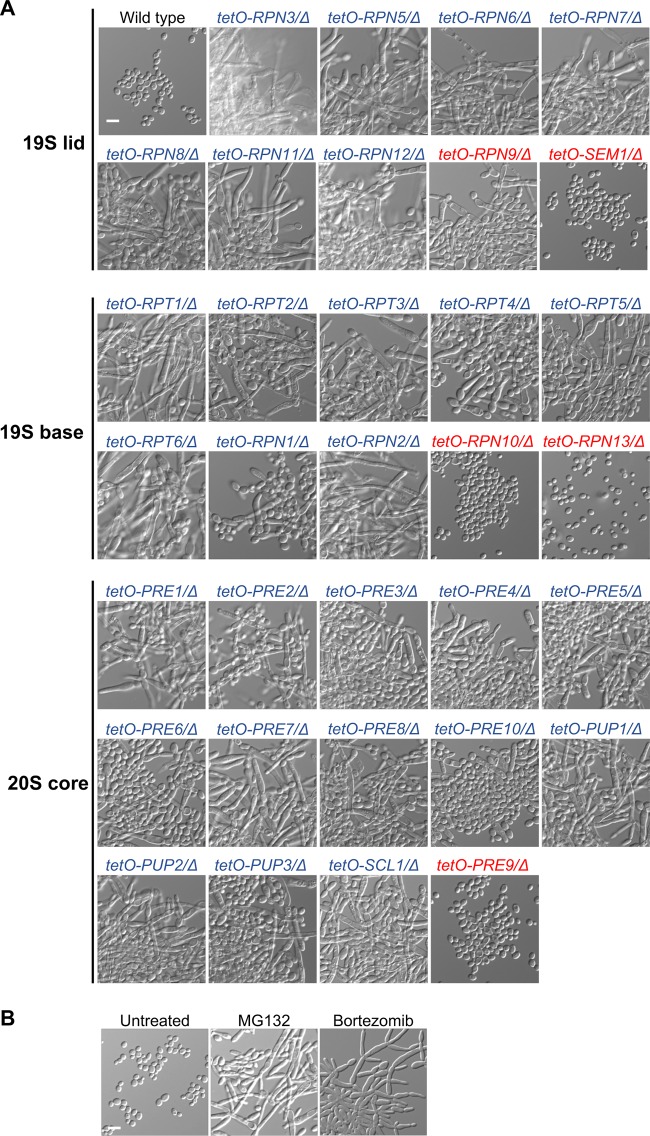
Inhibition of the proteasome induces C. albicans morphogenesis. (A) Genetic depletion of proteasome subunits of the 19S regulatory particle or 20S core particle induces morphogenesis in the absence of external inducing cues. Cells were grown in YPD medium in the presence of 0.05 μg/ml DOX at 30°C, with shaking, for 24 h to repress target gene expression. Strains were grown in batches with the wild-type control. Blue and red indicate proteasome subunit genes that are essential and nonessential for growth, respectively. (B) Pharmacological inhibition of the proteasome with two structurally distinct drugs, MG132 (200 μM) and bortezomib (520 μM), induces morphogenesis. Strains were grown in the absence or presence of drug at 30°C for 24 h under static conditions. Scale bar, 10 μm.

To characterize the impact of the proteasome on C. albicans growth and morphogenesis, we utilized gene replacement and conditional expression (GRACE) strains in which one allele of the gene of interest is deleted, and the only remaining allele is under the control of the tetracycline-repressible promoter, *tetO* (26). Repression of gene expression is achieved by the tetracycline analog doxycycline (DOX), which can be titrated to enable characterization of phenotypes associated with essential genes (27). A previous screen of a collection of GRACE strains provided an initial analysis of a subset of proteasome genes, identifying 24 as essential and 12 as repressors of morphogenesis (27). Here, we expanded the analysis to provide characterization of all 33 C. albicans proteasome subunit genes. Dose-response assays identified that 28 of the 33 proteasome subunit genes were essential for growth in rich medium following target gene repression with high concentrations of DOX (Fig. 1A; see also Fig. S1 in the supplemental material). *RPN9*, *SEM1*, *RPN10*, *RPN13*, and *PRE9* were not essential for growth even at concentrations of DOX at which significant transcriptional repression was achieved (Fig. S2A). To assess the impact of proteasome genes on morphogenesis, we grew the GRACE strains in rich medium in the presence of 0.05 μg/ml DOX, which was sufficient to repress target gene expression without substantial effects on growth (Fig. S1 and S2A). All strains grew as yeast in the absence of DOX (Fig. S3), as did the wild-type control in the absence or presence of DOX (Fig. 1A and Fig. S3), but transcriptional repression of 29 of the 33 proteasome subunits induced morphogenesis in the absence of an inducing cue, suggesting that they repress filamentation (Fig. 1A). Although depletion of *RPN9* induced filamentation, repression of the other four nonessential subunits, *SEM1*, *RPN10*, *RPN13*, and *PRE9*, did not induce robust filamentation even with higher concentrations of DOX (Fig. 1A and Fig. S2B). As a complementary approach, we pharmacologically inhibited proteasome function with two structurally distinct drugs, MG132 and bortezomib. Consistent with genetic depletion of the proteasome, we found that pharmacological inhibition of the proteasome induced C. albicans morphogenesis (Fig. 1B). Together, these observations demonstrate that proteasome function is essential for C. albicans growth and repression of the filamentation program.

10.1128/mBio.00290-20.1 FIG S1 Perturbation of the proteasome causes loss of viability. GRACE strains for proteasome subunit genes were grown in YPD medium in the presence of 5-fold dilutions of DOX starting at 20 μg/ml for 48 h at 30°C under static conditions. Growth was measured using OD_600_. Data shown are the average of two technical replicates, normalized relative to the no-drug control for the wild-type strain. Strains were assayed in batches with the wild-type control. Data were quantitatively displayed with color using Treeview (see color bar). After measurement of the growth, cultures were spotted onto drug-free YPD agar plates and grown at 30°C for 24 h to assess cidality. Genes were classified as essential if the relevant GRACE strain could not grow in the presence of 20 μg/ml of DOX. Blue and red indicate proteasome subunit genes that were identified as essential and nonessential for growth, respectively. Download FIG S1, PDF file, 0.6 MB. Copyright © 2020 Hossain et al. 2020 Hossain et al. This content is distributed under the terms of the Creative Commons Attribution 4.0 International license.

10.1128/mBio.00290-20.2 FIG S2 Perturbation of the proteasome causes accumulation of ubiquitinated proteins and affects proteasome function. (A) Depletion of proteasome gene expression is seen upon growth of the GRACE mutants in the presence of DOX. Cells were grown overnight in the absence of DOX (−) or in the presence of 0.05 μg/ml DOX (+) or 20 μg/ml DOX (++), as indicated, to repress target gene expression. Cells were subcultured into YPD medium under the same DOX conditions for 4 h before pelleting, RNA extraction, cDNA synthesis, and quantitative reverse transcription-PCR (qRT-PCR). Transcript levels were normalized to *ACT1* and *GPD1*. Significance was determined by unpaired *t* test. ****, *P* < 0.0001; ns indicates no significant difference (relative to results with the no-DOX control). (B) Genetic depletion of *RPN13*, *RPN10, PRE9*, or *SEM1* does not induce robust morphogenesis. Cells were grown in YPD in the absence of DOX (−) or presence of 20 μg/ml DOX (++) at 30°C, shaking, for 24 h to repress target gene expression. (C) Protein ubiquitination in response to transcriptional repression of proteasome subunit genes. Cells were grown under the same conditions as described for panel A to repress gene expression before protein extracts were prepared and analyzed by Western blotting with an anti-ubiquitin antibody. Immunoblot (upper) and Ponceau as a loading control (lower) are shown. (D) Dose-response assay with MG132. Cells were inoculated in YPD medium in the absence of DOX (−) or in the presence of 0.05 μg/ml DOX (+) or 20 μg/ml DOX (++) to repress target gene with 2-fold dilutions of MG132 ranging in concentration from 0 to 200 μM. After incubation at 30°C for 48 h under static conditions, growth was measured by the OD_600_. Data shown are the average of two technical replicates, normalized relative to level of the no-drug control for the wild-type strain. Data were visualized as described in the legend of Fig. S1. Download FIG S2, PDF file, 1.0 MB. Copyright © 2020 Hossain et al. 2020 Hossain et al. This content is distributed under the terms of the Creative Commons Attribution 4.0 International license.

10.1128/mBio.00290-20.3 FIG S3 GRACE strains of proteasome subunits grow in the yeast form in the absence of DOX. Cells were grown in YPD medium in the absence of DOX at 30°C, shaking, for 24 h. Strains were grown in batches with the wild-type control. Blue and red indicate proteasome subunit genes that are essential and nonessential for growth, respectively. Scale bar, 10 μm. Download FIG S3, PDF file, 1.9 MB. Copyright © 2020 Hossain et al. 2020 Hossain et al. This content is distributed under the terms of the Creative Commons Attribution 4.0 International license.

Next, we investigated the divergence in function of the nonessential proteasome genes and hypothesized that depletion of these subunits may not significantly affect proteasome function since their depletion did not impact growth. To assess this, we monitored the accumulation of ubiquitinated proteins by western blotting upon transcriptional repression of *RPN9*, *RPN13*, *RPN10*, *PRE9*, and *SEM1* and the essential subunit *RPT5.* As a complementary approach, we monitored sensitivity to MG132 as genetic perturbation of proteasome function should cause hypersensitivity to proteasome inhibition. As expected, transcriptional repression of *RPT5* impaired proteasome function, causing an accumulation of ubiquitinated proteins (Fig. S2C) and rendering cells hypersensitive to MG132 (Fig. S2D). Intriguingly, the impact of the nonessential subunits on proteasome function tracked with their effect on filamentation. *RPN9* depletion affected proteasome function to an intermediate level, leading to accumulated ubiquitinated proteins, hypersensitivity to MG132, and filamentation (Fig. 1 and Fig. S2C and D), while *RPN13* and *SEM1* depletion had no substantial effect on proteasome function or morphogenesis (Fig. S2B to D). *PRE9* and *RPN10* depletion led to slight accumulation of ubiquitinated proteins and hypersensitivity to MG132 (Fig. S2C and D), consistent with modest filamentation observed with high concentrations of DOX (Fig. S2B). This is in accordance with findings in S. cerevisiae that these five subunits, located in different proteasome subcompartments, are nonessential but play various roles in proteasome function (28–36). Overall, our work implicates the proteasome as a negative regulator of morphogenesis in C. albicans, which is uncoupled from the proteasome’s role in enabling growth.

### Filaments formed in response to proteasome inhibition and Hsp90 inhibition share structural similarities.

**FIG 2:**
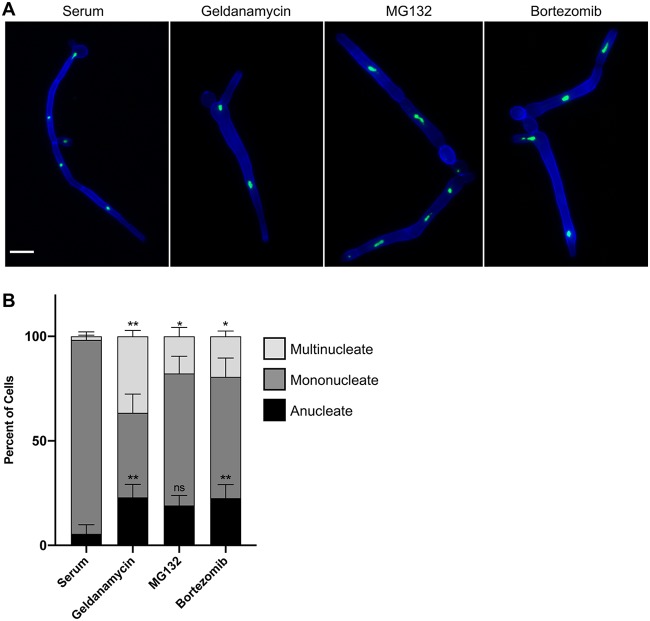
Filaments formed in response to proteasome inhibition and Hsp90 inhibition share structural similarities. (A) Filaments induced by proteasome inhibition are similar to filaments induced by Hsp90 inhibition but distinct from those induced by serum. Cells were grown under shaking conditions in YPD medium with 10% serum at 37°C for 4 h or in YPD medium with 10 μM geldanamycin, 200 μM MG132, or 520 μM bortezomib at 30°C for 7 h. Cell walls and septa were visualized using calcofluor white (blue), and the nuclei were visualized using a strain with the nucleolar protein, Nop1, tagged with GFP (green). Scale bar, 10 μm. (B) Quantification of the number of nuclei per cellular compartment. The numbers of nuclei in at least 100 cellular compartments were counted for each condition for three biological replicates. Means are graphed with the error bars displaying standard deviations. A paired *t* test was used to determine significant differences in percentages of multinucleate cells and anucleate cells in response to Hsp90 and proteasome inhibition relative to values for serum-induced filaments. *, *P* ≤ 0.05; **, *P* ≤ 0.01; ns, not significant.

To characterize the filaments induced by proteasome inhibition, we used a C. albicans strain expressing the green fluorescent protein (GFP)-tagged nucleolar protein Nop1 as a proxy for nuclear localization and stained the septa and cell walls with calcofluor white (37). As expected, filaments induced with the canonical filament-inducing cue of 10% serum at 37°C had distinct, mononucleate cellular compartments with parallel cell walls and regularly spaced septa while filaments induced by Hsp90 inhibition with geldanamycin had cell compartments with variable widths, sporadically spaced septa, and aberrant nuclear content (13) (Fig. 2A). Remarkably, filaments induced by proteasome inhibition with MG132 or bortezomib closely resembled filaments induced by Hsp90 inhibition (Fig. 2A), displaying less-defined compartments of variable widths, inconsistently spaced septa, and many anucleate or multinucleate compartments (Fig. 2A). Quantification of the number of nuclei per cellular compartment confirmed that proteasome and Hsp90 inhibition causes a significant increase in multinucleate cells compared to levels of serum-induced filaments, as well as an increase in anucleate cells (Fig. 2B). Together, these observations demonstrate that perturbation of proteostasis through Hsp90 or proteasome inhibition causes similar cell biological consequences, suggesting functional relationships between these filamentation programs.

### cAMP-PKA signaling is required for filamentation in response to proteasome inhibition.

**FIG 3:**
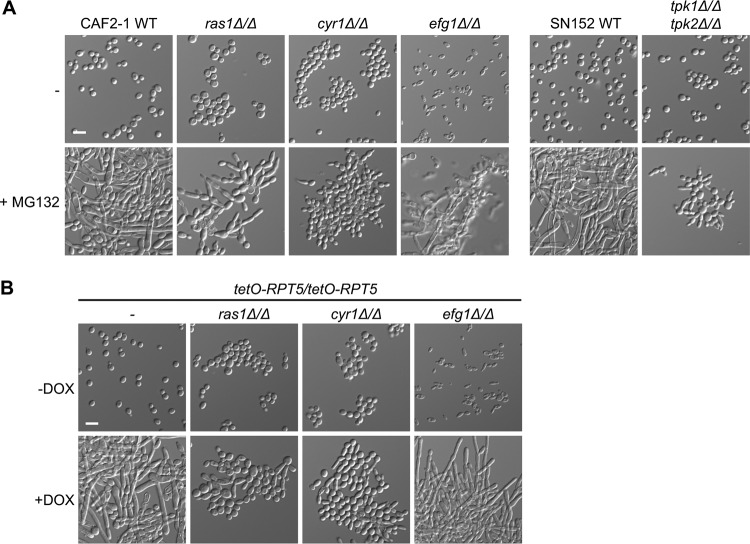
cAMP-PKA signaling is necessary for filamentation in response to proteasomal inhibition. (A) Filamentation induced by proteasome inhibition requires Ras1, Cyr1, and the catalytic subunits of PKA, Tpk1 and Tpk2, but not the transcription factor Efg1. Cells were grown in YPD medium in the absence or presence of 200 μM MG132 for 48 h at 30°C under static conditions prior to imaging. (B) Filamentation induced by depletion of *RPT5* requires Ras1 and Cyr1 but not Efg1. To repress *RPT5* expression, cells were grown overnight at 30°C in YPD medium in the absence or presence of 0.05 μg/ml DOX with shaking and subsequently grown in the absence or presence of 0.25 μg/ml DOX for 48 h at 30°C under static conditions prior to imaging. Scale bar, 10 μm. WT, wild type.

Given the morphological similarities between filaments induced by Hsp90 and proteasome inhibition, we hypothesized that there could be functional relationships between how these regulators influence morphogenesis. To assess this, we monitored whether the same genetic circuitry enables filamentation in response to inhibition of Hsp90 and the proteasome. We investigated the cAMP-PKA signaling cascade, which is important for morphogenesis in response to diverse cues (9, 14). Filamentation induced by Hsp90 inhibition requires the GTPase Ras1, the adenylyl cyclase Cyr1, and the catalytic subunits of PKA, Tpk1 and Tpk2, but not the transcription factor Efg1, a critical morphogenetic regulator which is activated by PKA (14). As with filamentation induced by Hsp90 inhibition, we found that Ras1, Cyr1, Tpk1, and Tpk2 are necessary for filamentation in response to proteasome inhibition with MG132 while Efg1 is dispensable (Fig. 3A). As a complementary approach, we generated tetracycline-repressible strains to genetically deplete *RPT5* and perturb proteasome function in the cAMP-PKA mutants. Consistent with MG132 treatment, filamentation induced by genetic depletion of *RPT5* was contingent on Ras1 and Cyr1 but independent of Efg1 (Fig. 3B). Genetic depletion of *RPT5* was sufficient to perturb proteasome function, as evidenced by lethality at high DOX concentrations (Fig. S4). We also found that Tec1, Bcr1, Flo8, Cek1, Cph1, and Ace2 were dispensable for filamentation in response to Hsp90 or proteasome inhibition (Fig. S5), consistent with our model that these perturbations to proteostasis induce morphogenesis through common genetic circuitry. This highlights that Hsp90 and the proteasome regulate morphogenesis through cAMP-PKA signaling and implicates additional downstream factors in activation of transcriptional programs governing morphogenesis in response to perturbation of proteostasis.

10.1128/mBio.00290-20.4 FIG S4 Perturbation of the proteasome results in loss of viability in cAMP-PKA pathway mutants. Dose-response assay with DOX. Cells were grown overnight in YPD medium in the absence and presence of 0.05 μg/ml DOX to repress *RPT5* expression. Cells were inoculated with 5-fold dilutions of DOX starting at 20 μg/ml. After growth for 48 h at 30°C under static conditions, growth was measured using the OD_600_. Data shown are the average of two technical replicates, normalized relative to the level of the no-drug control for the wild-type strain. Data were visualized as described in the legend of Fig. S1. After measurement of growth, cultures were spotted onto drug-free YPD agar plates and allowed to grow at 30°C for 24 h to assess cidality. Download FIG S4, PDF file, 0.4 MB. Copyright © 2020 Hossain et al. 2020 Hossain et al. This content is distributed under the terms of the Creative Commons Attribution 4.0 International license.

10.1128/mBio.00290-20.5 FIG S5 Many positive regulators of morphogenesis are dispensable for filamentation in response to Hsp90 and proteasome inhibition. Mutants of positive regulators of morphogenesis and their respective wild-type strains are capable of filamentation in response to proteasome and Hsp90 inhibition. Cells were grown in YPD medium (−) with 200 μM MG132 or 5 μM geldanamycin for 24 h at 30°C under static conditions before imaging. Scale bar, 10 μm. Download FIG S5, PDF file, 1.6 MB. Copyright © 2020 Hossain et al. 2020 Hossain et al. This content is distributed under the terms of the Creative Commons Attribution 4.0 International license.

### Proteasome inhibition overwhelms Hsp90’s functional capacity.

Proteasome inhibition results in global defects in ubiquitin turnover and an accumulation of ubiquitinated, misfolded proteins that cannot be degraded (38). Global protein misfolding can also be induced by stress, such as thermal insults, that overwhelms the functional capacity of Hsp90 as misfolded proteins can compete with clients for chaperone binding (39, 40). Hsf1 is activated in response to stresses that perturb proteostasis, thereby inducing a heat shock response (HSR), which increases production of heat shock proteins (HSPs), such as Hsp90, Hsp70, and Hsp104 that facilitate the repair or degradation of damaged proteins (41). We hypothesized that proteasome inhibition induces filamentation by causing an accumulation of misfolded proteins that overwhelm Hsp90’s functional capacity, relieving repression on cAMP-PKA signaling.

**FIG 4:**
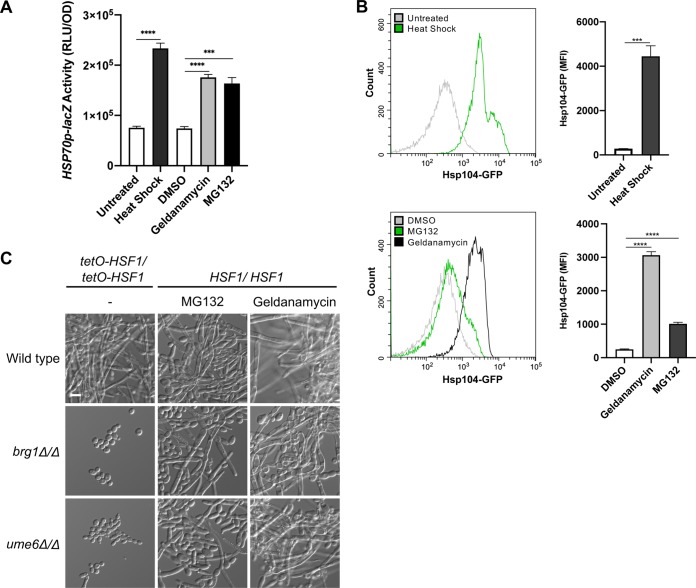
Proteasome inhibition overwhelms Hsp90’s functional capacity. (A) Induction of the heat shock response was monitored using an *HSP70p-lacZ* reporter strain. β-Galactosidase reporter activity was measured in relative luminescence units (RLU) after incubation of cells in YPD medium with shaking at 30°C for 5 h with or without 10 μM geldanamycin, 40 μM MG132, or 0.2% DMSO as a vehicle control. Heat shock was induced by growth at 42°C for 1 h. Each bar depicts the mean of technical triplicates, and all data were normalized to the OD_600_. Error bars display standard deviations. ***, *P* ≤ 0.001; ****, *P* ≤ 0.0001. (B) Activation of the heat shock response was monitored by measuring the induction of Hsp104-GFP protein expression. Mean GFP fluorescence intensities (MFI) of ∼20,000 cells were measured by flow cytometry after incubation of cells under the same growth conditions as described above. Representative flow cytometry histograms depict green fluorescence of gated events. In the quantification of the flow cytometry data, each bar depicts the mean MFI of technical triplicates. Error bars display standard deviations. ***, *P* ≤ 0.001; ****, *P* ≤ 0.0001. (C) Brg1 and Ume6 are dispensable for filamentation in response to proteasome and Hsp90 inhibition but are required for filamentation in response to *HSF1* overexpression. Cells were grown with 200 μM MG132 or 5 μM geldanamycin for 24 h at 30°C under static conditions. Overexpression of *HSF1* from the *tetO* promoter was achieved by growing cells in the absence of DOX for 24 h at 30°C under static conditions. Scale bar, 10 μm.

To determine if proteasome inhibition impairs Hsp90’s function, we monitored activation of the HSR as Hsp90 exerts a repressive effect on Hsf1 such that compromised Hsp90 function activates Hsf1 and induces the HSR (14, 39). To assess activation of the HSR as a measure of Hsp90 function in C. albicans, we utilized a reporter with the *HSP70* promoter fused to *lacZ*, integrated at the native *HSP70* locus (14). The *HSP70* promoter contains multiple heat shock elements allowing Hsf1 to bind and regulate *HSP70* expression under constitutive and heat stress conditions (42). We monitored *lacZ* activity as a measure of the HSR. As a control, we subjected cells to heat stress, which induced expression from the *HSP70* promoter, thus increasing reporter activity (Fig. 4A). Proteasome inhibition also significantly induced *lacZ* activity, phenocopying Hsp90 inhibition (Fig. 4A). As an alternative approach, we monitored the induction of an additional Hsf1 target, *HSP104*, using a C. albicans strain expressing GFP-tagged Hsp104 from its native promoter. Using flow cytometry, we found that both heat shock and Hsp90 inhibition induced Hsp104-GFP levels (Fig. 4B). Consistent with our model, Hsp104-GFP levels increased significantly in response to proteasome inhibition (Fig. 4B). Together, these observations demonstrate that inhibiting the proteasome compromises Hsp90’s capacity to repress Hsf1 activation.

An additional complexity in assessing if proteasome inhibition induces filamentation via compromise of Hsp90 function is that Hsf1 also influences morphogenesis. *HSF1* overexpression induces C. albicans filamentation by directly activating the expression of positive regulators of filamentation, including *BRG1* and *UME6* (17). To determine if the proteasome induces morphogenesis through Hsf1 activation, we determined whether filamentation induced by proteasome inhibition and *HSF1* overexpression requires the same genetic circuitry. While Brg1 and Ume6 are essential for filamentation in response to *HSF1* overexpression, both regulators were dispensable for filamentation in response to proteasome inhibition (Fig. 4C) and Hsp90 inhibition (12, 43) (Fig. 4C). Consistent with this finding, *HSF1* overexpression requires signaling through Efg1 but not Ras1 to induce morphogenesis (17), unlike inhibition of Hsp90 or the proteasome, suggesting distinct genetic dependencies for Hsf1. Overall, our findings suggest that inhibition of the proteasome results in an accumulation of ubiquitinated, misfolded proteins which overwhelm the functional capacity of Hsp90, relieving Hsp90-mediated repression of cAMP-PKA signaling to induce morphogenesis.

### The proteasome represses morphogenesis in diverse fungal species.

**FIG 5:**
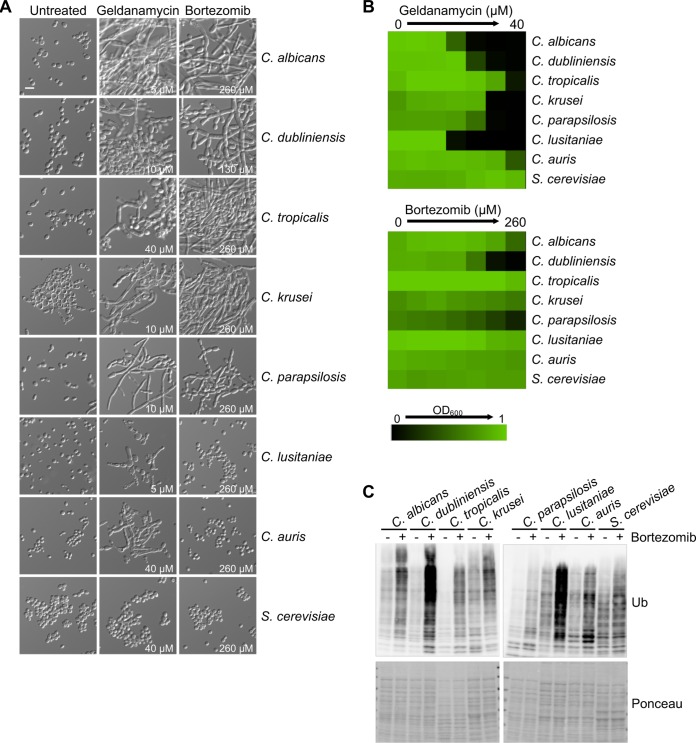
Proteasome inhibition induces morphogenesis in diverse fungal species. (A) Pharmacological inhibition of the proteasome with bortezomib induces morphogenesis in C. albicans, C. dubliniensis, C. tropicalis, C. krusei, and C. parapsilosis. Hsp90 inhibition with geldanamycin induces morphogenesis in all *Candida* species tested. Strains were grown in the absence or presence of the indicated concentrations of drug at 30°C for 24 h under static conditions before imaging. Scale bar, 10 μm. (B) Dose-response assays with geldanamycin or bortezomib. Cells were inoculated in YPD medium with 2-fold dilutions of geldanamycin or bortezomib. After incubation at 30°C for 24 h under static conditions, growth was measured by the OD_600_. Data shown are the average of two technical replicates. Data are quantitatively displayed with color using Treeview (as indicated by the legend). (C) Protein ubiquitination in response to inhibition of the proteasome. Cells were grown for 7 h in the absence or presence of bortezomib (treated with the same concentrations as described for panel A) at 30°C, with shaking. Protein extracts were prepared and analyzed by western blotting. Immunoblots with an anti-ubiquitin antibody (Ub) and Ponceau as a loading control are shown.

To determine whether filamentation in response to proteasome inhibition is a conserved fungal response, we assessed the effect of proteasome inhibition on the morphology of seven additional filamentation-competent fungal species: Candida dubliniensis, Candida tropicalis, Candida krusei, Candida parapsilosis, Candida lusitaniae, Candida auris, and S. cerevisiae Σ1278b. We observed that morphogenesis was induced to various extents in C. albicans, C. dubliniensis, C. tropicalis, C. krusei, and C. parapsilosis in response to proteasome inhibition while no filaments were observed in C. lusitaniae, C. auris, or S. cerevisiae, even at the highest bortezomib concentrations tested (Fig. 5A and B). Bortezomib treatment resulted in a detectable increase in ubiquitinated proteins in all species tested, confirming that our treatment conditions were sufficient to perturb the proteasome in all species (Fig. 5C). Notably, each species accumulated ubiquitinated proteins to different extents, which did not track with susceptibility to bortezomib or the ability to undergo morphogenesis (Fig. 5A to C). For example, C. tropicalis had modest accumulation of ubiquitinated proteins in response to bortezomib and substantial filamentation while C. lusitaniae had robust accumulation of ubiquitinated proteins despite no filamentation. This suggests that accumulation of ubiquitinated proteins is insufficient to drive filamentation in some fungal species. Intriguingly, all *Candida* species tested underwent filamentation in response to Hsp90 inhibition, including C. lusitaniae and C. auris, which remained as yeast in response to proteasome inhibition (Fig. 5A). This finding highlights either that there are distinct thresholds for the levels of inhibition of different facets of proteostasis required to induce filamentation across the diverse species or that evolutionary divergence has occurred in cellular pathways governing these morphogenetic programs. There is precedence for the evolutionary divergence of cellular responses to the accumulation of aggregation-prone proteins as C. albicans has been found to be highly resistant to toxicity induced by polyglutamine (polyQ) proteins compared to resistance of S. cerevisiae, despite similar levels of polyQ proteins in the proteome (44). Overall, we identified a role of the proteasome in regulating fungal morphogenesis that is conserved across diverse fungal species.

## DISCUSSION

**FIG 6:**
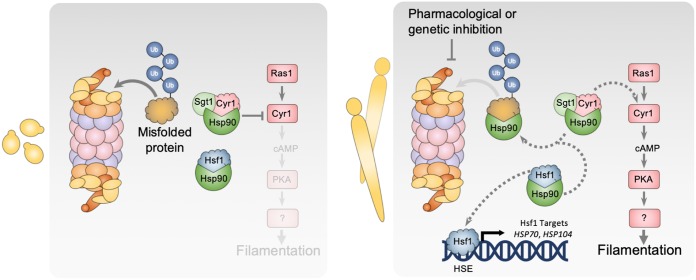
Model depicting the role of the proteasome in governing morphogenesis. Hsp90 and its cochaperone Sgt1 physically interact with Cyr1, keeping it in an inactive conformation, repressing the cAMP-PKA signaling cascade. Hsp90 also binds Hsf1, which represses Hsf1 function. Inhibition of the proteasome leads to an accumulation of ubiquitinated, misfolded proteins that overwhelm the functional capacity of Hsp90, thereby relieving the repression on Hsf1 and the cAMP-PKA pathway, inducing the HSR and morphogenesis, respectively. The proteasome could also affect the cAMP-PKA pathway through additional mechanisms that are independent of Hsp90.

Protein chaperone systems and the ubiquitin-proteasome pathway are key modulators of protein homeostasis and cellular signaling, with a profound impact on survival and proliferation of all eukaryotes. Here, we further establish the critical role of proteostasis in regulating fungal morphogenesis, a key virulence trait. We implicate the proteasome in repressing filamentation of diverse fungi such that pharmacological inhibition or genetic depletion of the proteasome induces filamentation in the absence of any external cue (Fig. 1). Filaments formed in response to proteasome or Hsp90 inhibition share structural similarities, depend on the same components of the cAMP-PKA signaling pathway, and activate the evolutionarily conserved heat shock response, suggesting a functional relationship between these filamentation programs (Fig. 2 to 4). Together, our findings are consistent with the model that proteasome inhibition results in an accumulation of misfolded, ubiquitinated proteins that overwhelm Hsp90 function, thereby relieving Hsp90-mediated repression of Hsf1 to activate the HSR and repression of the cAMP-PKA pathway to induce filamentation (Fig. 6).

Morphogenesis in response to pharmacological inhibition or genetic depletion of proteasome function is mediated through cAMP-PKA signaling, a key hub for C. albicans morphogenesis (Fig. 3). This observation supports findings of a functional connection between the proteasome and cAMP-PKA signaling in fungi and mammalian cells, with profound effects on disease. For C. neoformans, increased PKA signaling influences virulence by inducing production of an enlarged polysaccharide capsule, which contributes to immunomodulatory and antiphagocytic properties during infection (22, 45). Intriguingly, PKA signaling is suggested to balance proteasome levels to regulate capsule production as reduced proteasome function blocks capsule production (23). In addition, in mammalian cells increased intracellular cAMP stimulates PKA and activates the peptidase of the proteasome via phosphorylation of the 19S subunit, Rpn6 (46). Increased PKA signaling leads to the degradation and clearance of aggregation-prone proteins such as tau, which accumulates in brains marked by neurodegenerative diseases (46). Interestingly, activation of the cAMP-PKA pathway also promotes the ubiquitination and proteolysis of catalytic subunits of PKA via the proteasome, attenuating downstream PKA signaling (47). Together, this suggests a conserved functional connection between the proteasome and PKA signaling, with consequences for cellular homeostasis and disease.

Our work contributes to a growing appreciation of the interconnected roles of the proteasome, Hsp90, and Hsf1 in maintaining global protein homeostasis during stress. Thermal insults or proteotoxic stresses cause a global accumulation of misfolded proteins, which are bound by Hsp90 and other chaperones to mediate refolding or targeted for degradation by the proteasome. Excessive misfolded proteins can overwhelm the functional capacity of Hsp90, causing the chaperone to be titrated away from its client proteins and relieving its repression on Hsf1 (39, 41). Consistent with these findings, our work demonstrates that the proteasome can also impact Hsp90 function. Proteasome inhibition halts the degradation of ubiquitinated, misfolded proteins, leading to their accumulation. We observed that these accumulated, damaged proteins overwhelm Hsp90’s functional capacity, activating the HSR. This supports evidence in both S. cerevisiae and mammalian cells that proteasome inhibition activates Hsf1 and the HSR to induce transcriptional changes that help restore proteostasis (48, 49). Activated Hsf1 induces the expression of chaperone targets, including *HSP90*, *HSP70*, and *HSP104*, which are upregulated to mediate refolding and prevent aggregation of misfolded proteins (49). Intriguingly, inhibition of Hsf1 also impacts Hsp90 function (17), likely due to the misregulation of chaperones and Hsp90 cochaperones required for proper protein folding and Hsp90 function, respectively. There are additional functional relationships between Hsp90 and the proteasome as S. cerevisiae Hsp90 has been found to assist in proteasome assembly and functional integrity (50). Further, destabilized Hsp90 client proteins are targets of the proteasome such that Hsp90 inhibition induces rapid degradation of client proteins via the ubiquitin-proteasome pathway (51, 52). Additionally, in C. albicans, Hsp90 inhibition increases the levels of 20S proteasome proteins (53), potentially to meet the global demand for proteolysis of misfolded Hsp90 clients. Together, these observations demonstrate a complex functional network of proteins acting in concert to maintain protein homeostasis whereby perturbation of any component leads to the induction of filamentation.

While our findings support a model of the proteasome impacting morphogenesis through Hsp90 (Fig. 6), it is also possible that filamentation is induced at least in part through effects on Hsf1 or directly through its role in regulating protein abundance. Upon activation, Hsf1 binds to an expanded set of target genes involved in filamentation (42), and Hsf1 overexpression can drive filamentation by directly upregulating positive regulators of morphogenesis (17). It is possible that proteasome inhibition, in addition to activating Hsf1, could drive Hsf1 to induce the expression of positive morphogenetic regulators, triggering filamentation. However, distinct genetic circuitry enables morphogenesis in response to proteasome inhibition and *HSF1* overexpression, implicating contributions independent of Hsf1 (Fig. 4C). Alternatively, the proteasome could regulate filamentation directly through the turnover of morphogenetic regulators. The ubiquitin-proteasome system is known to govern progression through the cell cycle through the regulated degradation of cell cycle proteins (54). Proteasome inhibition could cause the accumulation and increased stability of cyclins, halting cell cycle progression, which is sufficient to induce morphogenesis (9). This hypothesis is consistent with our observation that filaments formed in response to proteasome inhibition display aberrant nuclear contents (Fig. 2). Proteasome inhibition could also cause accumulation of other positive morphogenetic regulators that cannot be degraded. Overall, while our evidence supports a model in which proteasome inhibition drives morphogenesis primarily by impairment of Hsp90 function, multiple contributing factors could work in parallel to induce filamentation.

This work highlights that perturbation of proteostasis has profound effects on C. albicans morphogenetic transitions. In addition to the impact of the proteasome on morphogenesis, other regulators of the ubiquitin-proteasome complex also have been implicated in morphogenesis. Ubiquitin is a small protein that is covalently attached to substrates through the sequential activity of E1 ubiquitin-activating enzymes, E2 ubiquitin-conjugating enzymes, and E3 ubiquitin ligases, targeting them for proteasomal degradation. Deletion of the polyubiquitin-encoding gene *UBI4* causes a depletion of cellular ubiquitin (55), which impedes proteasome function and induces filamentation (56, 57). Filamentation is also induced upon impairment of the E2 ubiquitin conjugating enzyme *RAD6* (58) and members of the E3 ubiquitin ligase Skp, Cullin, F-box (SCF) complex, which catalyzes the ubiquitination of many cell cycle proteins for proteasomal degradation (59–61). Overall, this suggests that the ubiquitin-proteasome system plays a pivotal role in morphogenesis as disruption at any step of this pathway induces filamentation. Beyond the ubiquitin-proteasome pathway, it also remains to be understood if morphogenesis is influenced by other proteostasis pathways, such as translation, the unfolded protein response, or vacuolar degradation. We hypothesize that any perturbation of proteostasis that leads to aberrantly folded proteins could compromise Hsp90 function and induce morphogenesis.

The therapeutic potential of proteasome inhibition is appreciated for treating cancer, where bortezomib and other proteasome inhibitors are used routinely for treating multiple myeloma and mantle cell lymphoma (62). Beyond cancer treatment, our findings suggest that proteasome inhibitors could offer a promising mechanism for broadly impairing fungal virulence. Targeting fungal virulence traits offers a promising alternative strategy for disarming fungal pathogens during an age of rising antimicrobial drug resistance. Targeting virulence factors offers several advantages over conventional therapies, including expanding the repertoire of fungal targets, minimizing deleterious effects on the host mycobiome, and reducing selection for the evolution of resistance (63, 64). Targeting proteostasis signaling is an attractive approach to attenuating C. albicans virulence. In support of this, compromising the ubiquitin-proteasome system through deletion of *UBI4* was found to attenuate C. albicans virulence in a mouse model of infection (57). The challenge for exploiting the proteasome as an antifungal drug target is the requirement for fungus-selective molecules to selectively disrupt proteostasis in the pathogen and not the host. Structure-enabled approaches provide a powerful opportunity to develop species-selective inhibitors of core cellular regulators, thereby expanding the target space for antifungal drug development (65–67).

## MATERIALS AND METHODS

### Strains and culture conditions.

All strains and plasmids used in this study are listed in Tables S1 and S2, respectively, in the supplemental material. All oligonucleotide sequences used in this study are listed in Table S3. Strains were grown in YPD medium (1% yeast extract, 2% Bacto peptone, 2% glucose) at 30°C unless otherwise specified. For solid medium, 2% agar was added. Stocks of geldanamycin (G-4500 [LC Laboratories] or 16D07-MM [Invivogen]), MG132 (S2619; Selleck Chemicals), and bortezomib (B675700; Toronto Research Chemicals) were made in dimethyl sulfoxide (DMSO). Doxycycline (DB0889; BioBasic) stocks were made in water. Details of strain construction and supplemental methods are given in Text S1.

10.1128/mBio.00290-20.6 TEXT S1 Supplemental methods. Download Text S1, PDF file, 0.1 MB. Copyright © 2020 Hossain et al. 2020 Hossain et al. This content is distributed under the terms of the Creative Commons Attribution 4.0 International license.

10.1128/mBio.00290-20.7 TABLE S1 Strains used in this study. Download Table S1, PDF file, 0.1 MB. Copyright © 2020 Hossain et al. 2020 Hossain et al. This content is distributed under the terms of the Creative Commons Attribution 4.0 International license.

10.1128/mBio.00290-20.8 TABLE S2 Plasmids used in this study. Download Table S2, PDF file, 0.1 MB. Copyright © 2020 Hossain et al. 2020 Hossain et al. This content is distributed under the terms of the Creative Commons Attribution 4.0 International license.

10.1128/mBio.00290-20.9 TABLE S3 Oligonucleotides used in this study. Download Table S3, PDF file, 0.1 MB. Copyright © 2020 Hossain et al. 2020 Hossain et al. This content is distributed under the terms of the Creative Commons Attribution 4.0 International license.

### Dose-response assays.

Dose-response assays were performed in flat-bottom, 96-well microtiter plates (Sarstedt), as previously described (68). Assays were set up in a total volume of 0.1 ml/well with 2-fold dilutions of each drug. Cell densities of overnight cultures were determined, and ∼10^3^ cells were inoculated into each well. Plates were incubated at 30°C for 24 h before the optical density at 600 nm (OD_600_) was measured using a spectrophotometer (Molecular Devices). Each strain was tested in technical and biological duplicates. Data are quantitatively displayed with color using Java TreeView, version 1.1.6r4 (http://jtreeview.sourceforge.net).

### Microscopy.

For all microscopy, cells were imaged using differential interference contrast (DIC) microscopy with a Zeiss Axio Imager.MI (Carl Zeiss) and an X-cite series 120 light source for fluorescence. To monitor C. albicans filamentation in GRACE mutants, overnight cultures were diluted to an OD_600_ of 0.1 in YPD medium with or without 0.05 μg/ml DOX and grown overnight at 30°C with shaking before imaging. For *tetO-RPT5*/*tetO-RPT5* strains, overnight cultures were diluted to an OD_600_ of 0.1 to 0.3 and grown overnight with or without 0.05 μg/ml DOX with shaking. Subsequently ∼10^3^ cells were inoculated into wells of a 96-well plate with or without 0.25 μg/ml DOX and grown under static conditions for 48 h at 30°C before imaging.

For Nop1-GFP visualization, an overnight culture of strain CaLC4506 was diluted in YPD medium to an OD_600_ of 0.1 and grown with shaking for 4 h at 37°C with 10% heat-inactivated newborn calf serum (26010074; Gibco), or for 7 h at 30°C with 10 μM geldanamycin, 200 μM MG132, or 520 μM bortezomib. The cells were treated with 5 μg/ml calcofluor white (F3543; Sigma-Aldrich) dissolved in water. To visualize fluorescence, ET green fluorescent protein (GFP) and 4′,6-diamidino-2-phenylindole (DAPI) hybrid filter sets (Chroma Technology) were used. Quantification of nuclei was performed by counting the number of nuclei per cellular compartment in at least 100 compartments per condition for three biological replicates, using ImageJ software. Statistical significance was determined using a paired *t* test using GraphPad Prism, version 8. For all other filamentation assays, strains were grown overnight in YPD medium at 30°C with shaking, and ∼10^3^ cells were inoculated into wells of a 96-well plate with or without geldanamycin, bortezomib, or MG132 and grown for 24 h at 30°C under static conditions before imaging.

### β-Galactosidase assays.

Strain CaLC922, containing the *HSP70* promoter driving expression of the *lacZ* gene from Streptococcus thermophilus integrated at the *HSP70* native locus, was grown overnight in YPD medium with 80 μg/ml uridine at 30°C with shaking, diluted to an OD_600_ of 0.05, and grown with shaking for 5 h at 30°C with or without 10 μM geldanamycin, 40 μM MG132, or 0.2% DMSO as a vehicle control. To induce heat shock, overnight cultures were diluted to an OD_600_ of 0.1, grown for 4 h at 30°C before addition of an equal volume of fresh YPD medium at 54°C, and immediately transferred to 42°C for incubation for 60 min with shaking. The induction of galactosidase activity was measured using a luminescent Gal-Screen reagent (catalog no. T1030; Applied Biosystems) at a reagent/cell ratio of 1:1, as per the manufacturer’s recommendations, and normalized to cell growth measured by the OD_600_. Two independent experiments with three replicates were performed. Statistical significance was determined by averaging technical triplicates, and an unpaired *t* test was performed using GraphPad Prism, version 8.

### Flow cytometry.

To measure Hsp104-GFP levels, CaLC3067 was grown under the same conditions used in the β-galactosidase assays. Subcultures were diluted 1:4 in phosphate-buffered saline (PBS) and transferred into a flat-bottom, 96-well microtiter plate (Corning). Assay volumes were 250 μl/well. Hsp104-GFP fluorescence was measured using the fluorescein isothiocyanate (FITC) setting in a CytoFLEX flow cytometer (Beckman Coulter, Inc.) for at least 20,000 events per sample. Data were analyzed using CytExpert software. Events were gated to capture at least ∼85% of the events after particulates and cellular debris were discarded. Representative histograms for gated events are shown. Mean fluorescence intensities (MFIs) were measured for technical triplicates. Two independent experiments were performed. Statistical significance was determined using an unpaired *t* test using GraphPad Prism, version 8.

### Western blotting.

Western blotting was carried out as described in Text S1. Ubiquitin epitopes were detected using an anti-ubiquitin antibody (P4D1, RRID:AB_628423; diluted 1:2,000 [sc-8017; Santa Cruz Biotechnology]). Membranes were stained with 0.15% (wt/vol) Ponceau S (40% methanol, 15% acetic acid [P3504; Sigma]) as a loading control.
