# Supplementary figures and images for: The Proteasome Governs Fungal Morphogenesis via Functional Connections with Hsp90 and cAMP-Protein Kinase A Signaling
Source: mBio. 2020 Apr 21;11(2):e00290-20. doi: 10.1128/mBio.00290-20 (PMC7175089; doi:10.1128/mBio.00290-20)

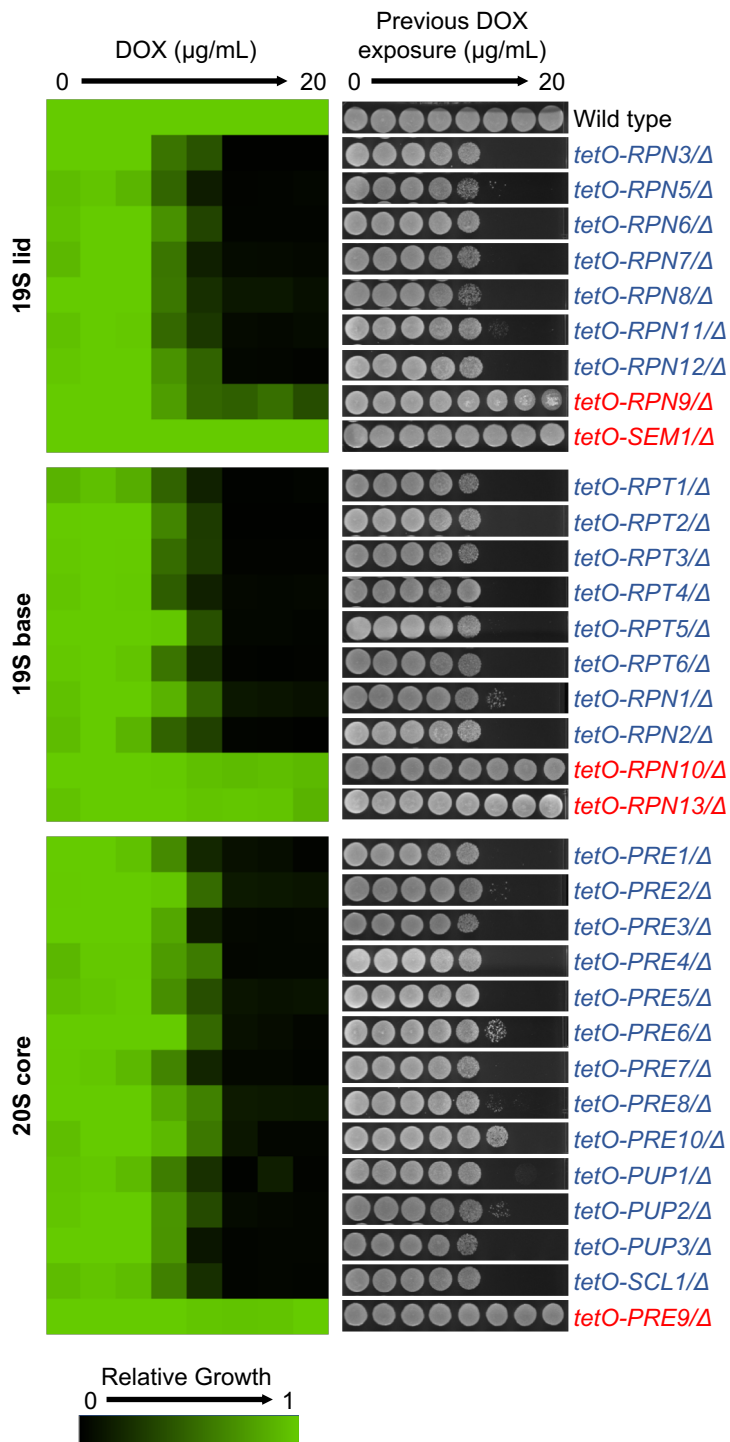

Supplement: FIG S1 [file mBio.00290-20-sf001.pdf]

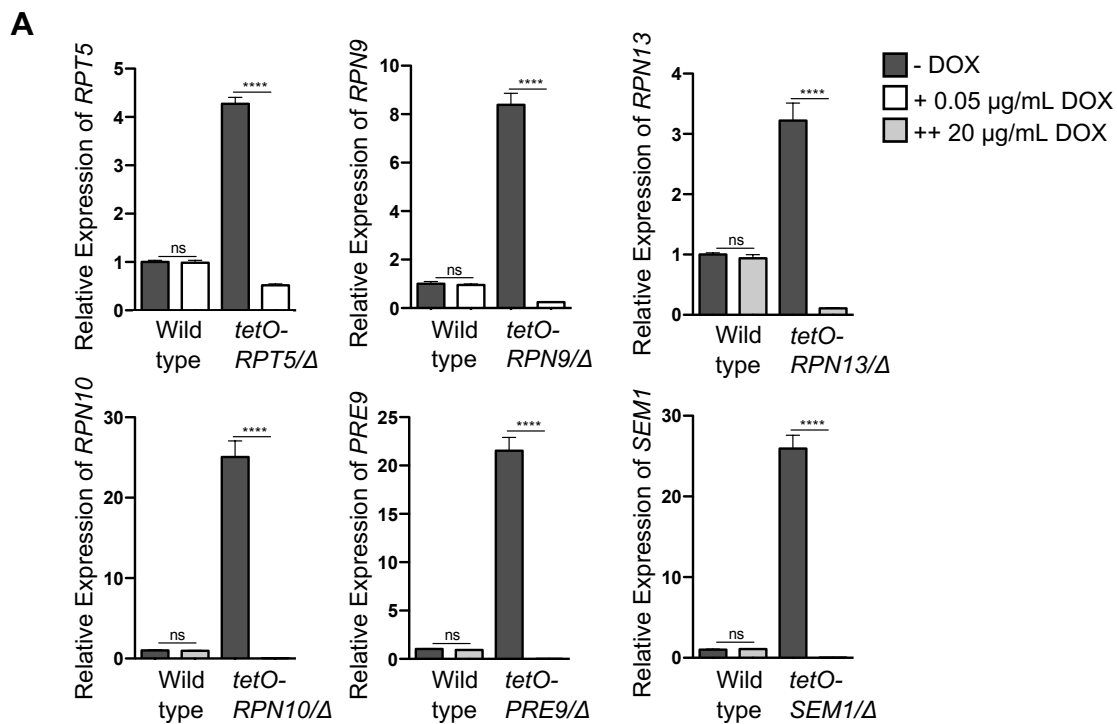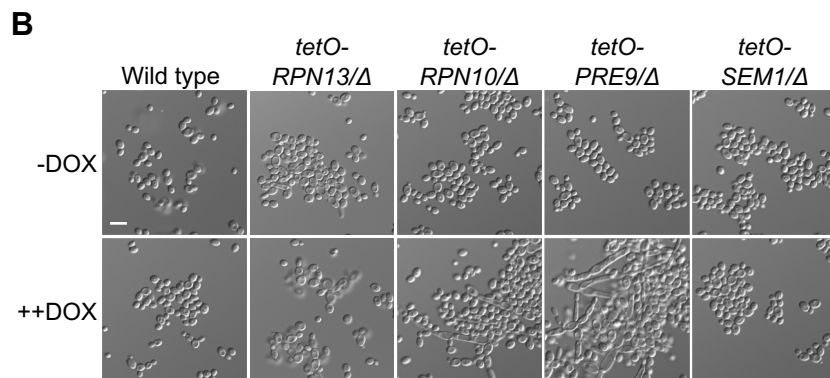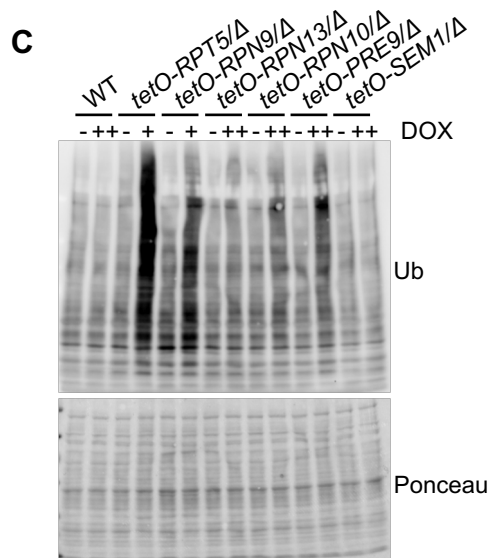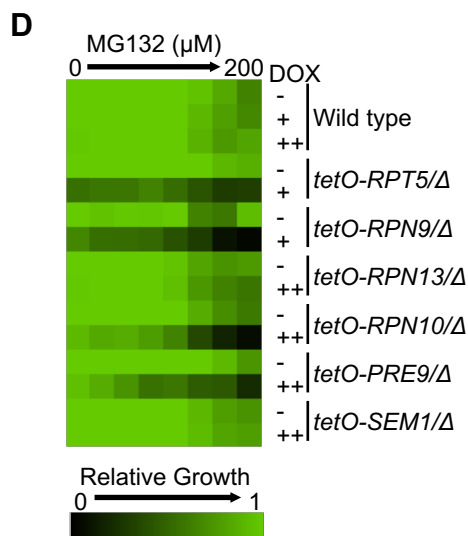

Supplement: FIG S2 [file mBio.00290-20-sf002.pdf]

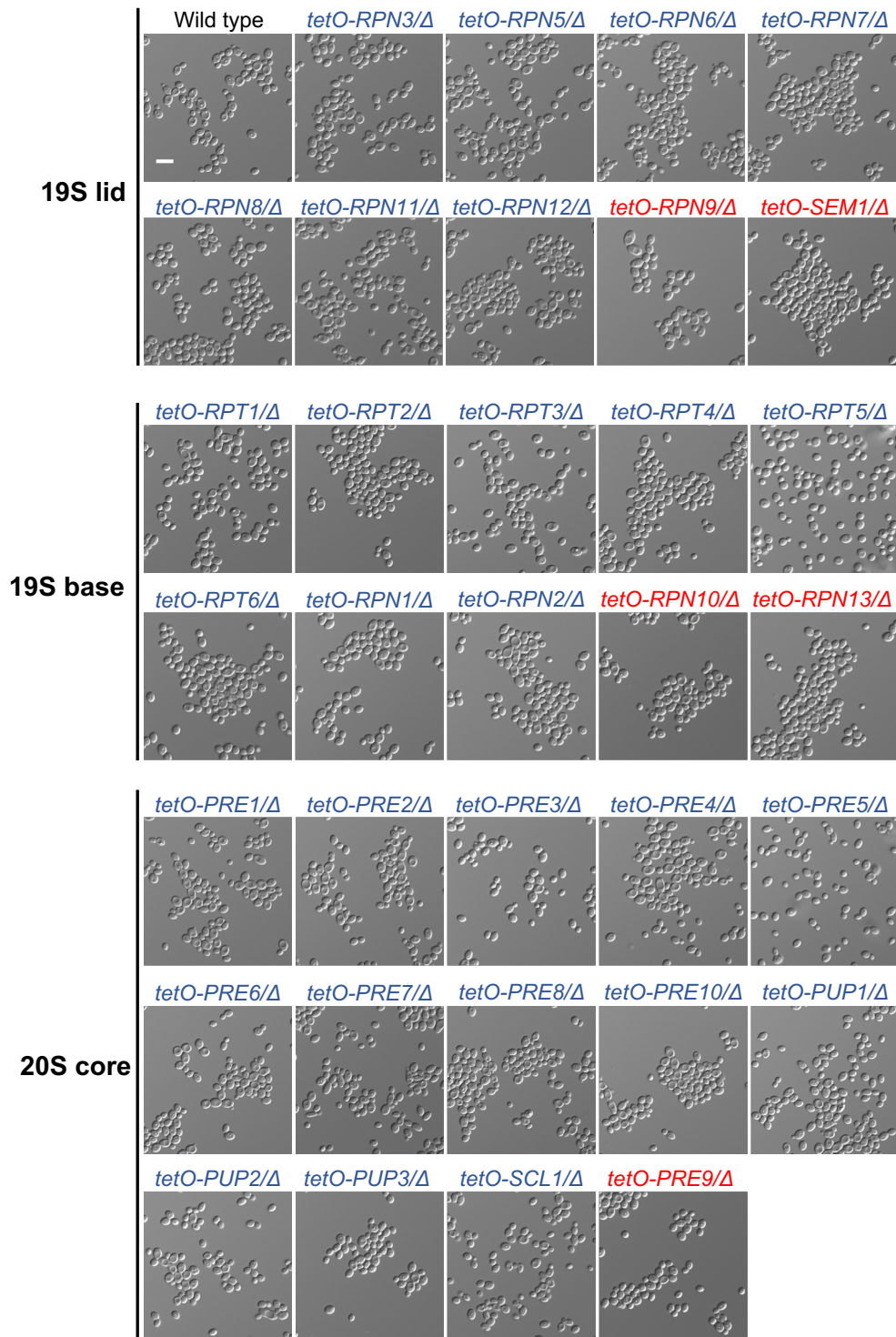

Supplement: FIG S3 [file mBio.00290-20-sf003.pdf]

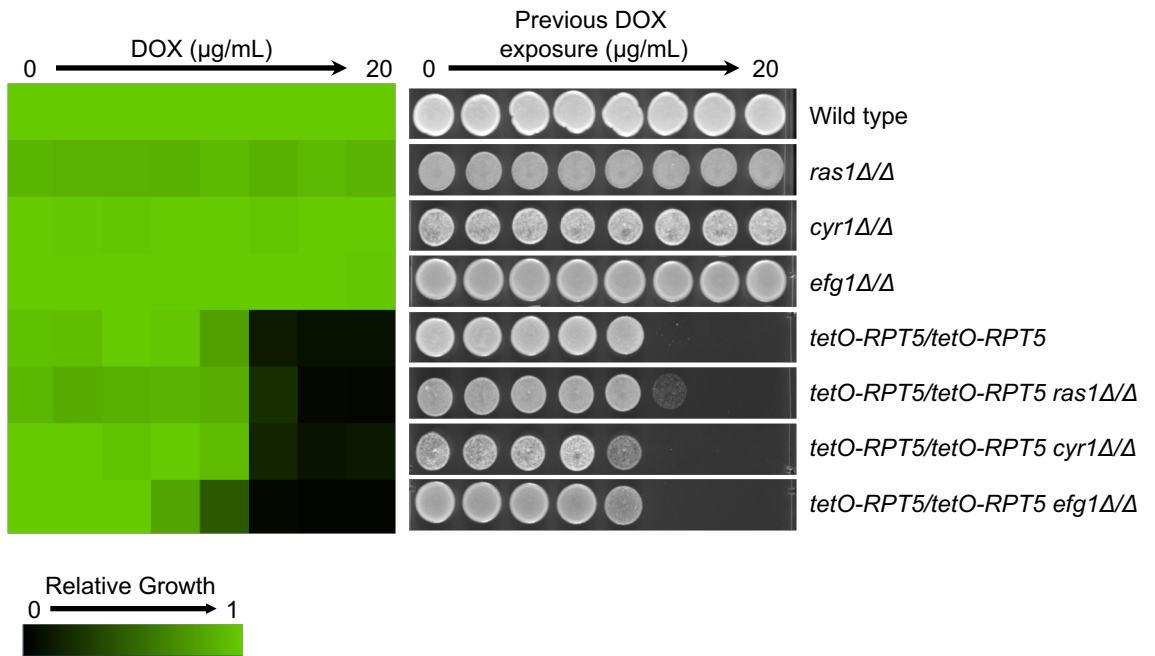

Supplement: FIG S4 [file mBio.00290-20-sf004.pdf]

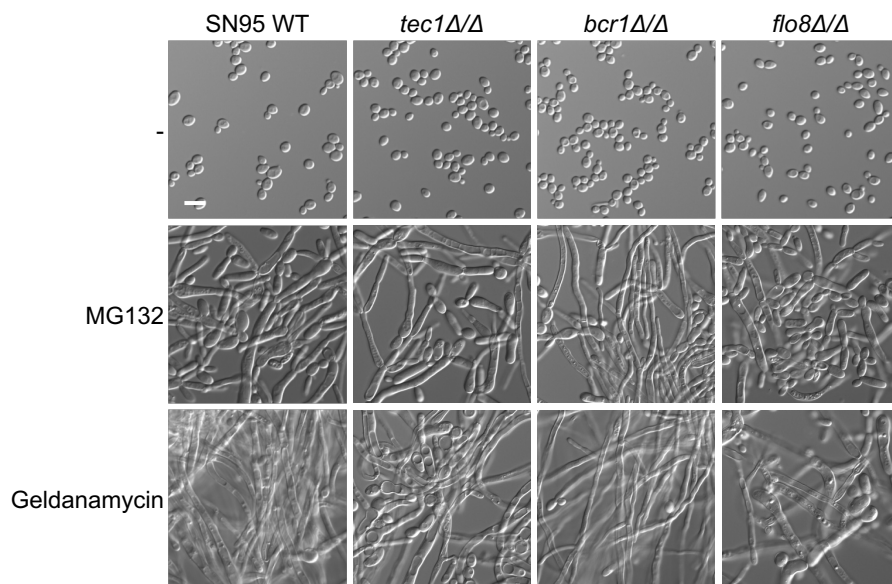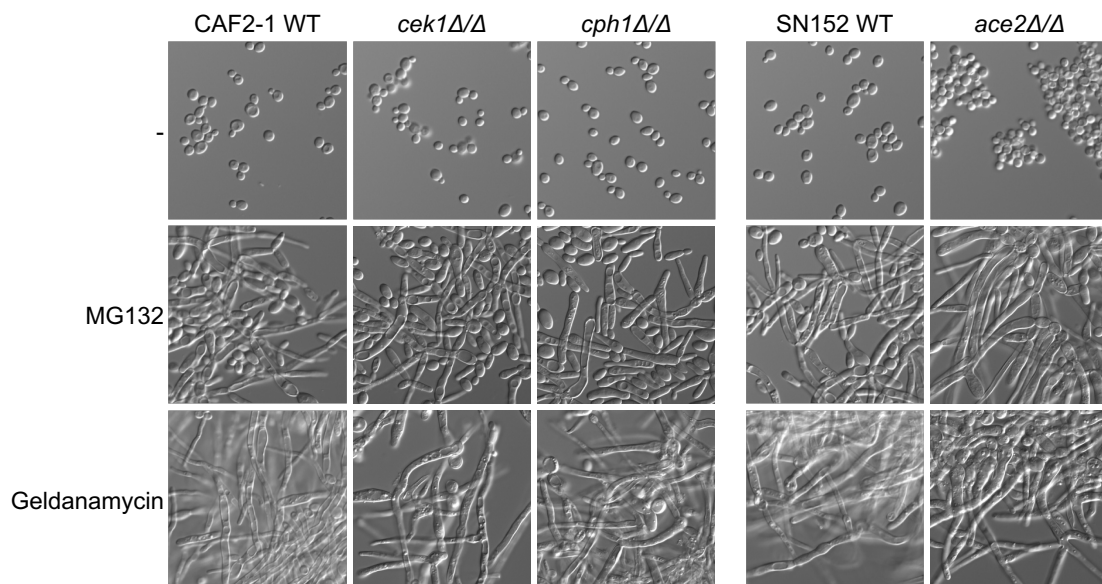

Supplement: FIG S5 [file mBio.00290-20-sf005.pdf]
