# Supplementary material for: The Proteasome Governs Fungal Morphogenesis via Functional Connections with Hsp90 and cAMP-Protein Kinase A Signaling
Source: mBio. 2020 Apr 21;11(2):e00290-20. doi: 10.1128/mBio.00290-20 (PMC7175089; doi:10.1128/mBio.00290-20)
Supplement: TEXT S1 [file mBio.00290-20-s0001.pdf]

Text S1: Supplemental methods

Strain construction:

To select for nourseothricin (NAT)-resistant mutants, nourseothricin (Jena Bioscience, 96736-11-7) was solubilized in water and supplemented into YPD agar plates (150 µg/mL to 250 µg/mL). Prototrophic colonies were selected on SD plates (0.674% yeast nitrogen base without amino acids with ammonium sulfate, 2% glucose and 2% agar).

GRACE strains: To expand the GRACE collection to have more comprehensive coverage of proteasome subunits, GRACE strains were generated from double-barcoded heterozygous (HET) mutants containing the tetracycline-repressible transactivator, as described previously with some modifications (1). Briefly, the NAT cassette and *tetO* promoter were PCR amplified from pLC763 using primers containing 20 to 22 base pairs complementary to the promoter replacement cassette and 70 base pairs of homology upstream or downstream of the start of the gene of interest (designated primer 1 and primer 2). This facilitates homologous recombination to insert the NAT and *tetO* promoter directly upstream of the start codon of the gene. When available, the NAT and *tetO* promoter construct were transformed in the relevant HET from the HET collection (2). NAT-resistant colonies were PCR tested for upstream integration of the *tetO* promoter using a primer that anneals ~250-350 base pairs upstream of the gene of interest (designated primer 3) and a primer that anneals within the NAT cassette (oLC6853), and/or downstream integration using a primer that anneals ~250-300 base pairs downstream of the start codon (designated primer 4) and a primer that anneals within the *tetO* cassette (oLC2535). NAT-resistant colonies were additionally tested by PCR to verify the absence of a wild-type allele of the target gene using primer 3 and primer 4.

Heterozygotes mutants for proteasome genes that were not available in the HET collection were constructed, as described previously with some modifications, before generating GRACE strains (1, 2). The *CaHIS3* cassette was PCR amplified from pLC1251 using primers containing 43 base pairs of homology at the 5′ or 3′ of the target gene, internal strain-identifying unique 20 base pair barcodes and flanking common primer sequences, and 18 base pairs which anneals to the 5′ or 3′ of the *CaHIS3* gene (designated primer 5 and primer 6). The cassette was transformed into the parent strain for the library, CaLC6106. Prototrophic colonies were PCR tested for upstream integration of the *CaHIS3* using primer 3 and a primer that anneals within the *HIS3* cassette (oLC6701). All primer sequences are provided in Table S3.

CaLC6152: The *tetO-RPT5/tetO-RPT5* strain was made using a transient CRISPR approach adapted from Min and colleagues (3). The promoter replacement cassette was PCR amplified from pLC605 using oLC7838 and oLC7839. The *CaCAS9* cassette was amplified from pLC963 using oLC6924 and oLC6925. The sgRNA fusion cassette was made by PCR amplifying from pLC963 with oLC5978 and oLC7843 (fragment A) and oLC5980 and oLC7842 (fragment B), and then fusion PCR was performed on the fragments using the nested primers oLC5979 and oLC5981. The *NAT-tetO* cassette, sgRNA, and Cas9 DNA were transformed into CaLC2724. Upstream integration was PCR tested using oLC534 and oLC7840, and downstream integration was tested using oLC4714 and oLC7841. Lack of a wild-type allele was PCR tested using oLC7840 and oLC7841. The NAT marker was excised by growth in YNB-BSA (0.17% yeast nitrogen base, 0.4% bovine serum albumin, 0.2% yeast extract and 2% maltose) to induce the FLP recombinase.

CaLC6153: The *tetO-RPT5/tetO-RPT5 efg1Δ/efg1Δ* strain was constructed using the same method as CaLC6152, except that the *tetO* cassette was transformed into CaLC563 (*efg1Δ/efg1Δ*).

CaLC6196: The *tetO-RPT5/tetO-RPT5 ras1Δ/ras1Δ* strain was constructed using the same method as CaLC6152, except that the *tetO* cassette was transformed into CaLC564 (*ras1Δ/ras1Δ*).

CaLC6227: The *tetO-RPT5/tetO-RPT5 cyr1Δ/cyr1Δ* strain was constructed using the same method as CaLC6152, except that the *tetO* cassette was transformed into CaLC555 (*cyr1Δ/cyr1Δ*).

Plasmid construction

pLC763: The plasmid pLC763 was derived from the plasmid pLC605 (4) by digesting pLC605 with ApaI to release the transactivator upstream of the *tetO* promoter.

pLC1251: *C. albicans HIS3* was amplified from genomic DNA from a HET strain (2) using primers oLC7860 and oLC7861. This amplicon and pLC1237 (pUC19) were digested with BamHI overnight and ligated.

Additional methods:

Quantitative reverse transcription PCR (qRT-PCR)

qRT-PCR was utilized to monitor target gene repression upon treatment with DOX. Overnights of strains were subcultured ±0.05 μg/mL or 20 μg/mL DOX overnight, as indicated. The next day, strains were subcultured into the same conditions and grown to mid-log phase. Cells were pelleted at 3000 rpm at 4°C, washed with cold, distilled water, and then flash frozen on a dry ice ethanol bath or liquid nitrogen before storing pellets at -80°C. Cells were lysed by bead beating with acid washed glass beads (Sigma G8772-500g) with the MiniBeadBeater-16 (BioSpec Products) six times for 30 seconds, with one minute on ice in between. RNA was extracted from the lysed cells using the QIAGEN RNeasy kit and DNase treated using the QIAGEN RNase free

DNase Set. Complementary DNA synthesis was performed using the iScript cDNA Synthesis Kit, utilizing both oligo (dT) and random primers for amplification. qRT-PCR was performed using the BioRad CFX-384 Real Time System in a 384 well plate, utilizing the Applied Biosystems Fast SYBR Green Master Mix with 10 µL reaction volume. Amplification was performed with the following cycle conditions: 5°C for 3 minutes, then 95°C for 10 seconds and 60°C for 30 seconds, for 40 cycles. The melt curve was completed with the following cycle conditions: 95°C for 10 seconds and 65°C for 10 seconds with an increase of 0.5°C per cycle up to 95°C. Reactions were performed with technical triplicates and data were analyzed using the BioRad CFX Maestro software. Statistical significance was determined using unpaired t-test with GraphPad Prism 8. The primer sequences used for monitoring expression of *ACT1* (oLC2285/oLC2286), *GPD1* (oLC752/oLC753), *RPN9* (oLC8159/oLC8161), *RPN13* (oLC8121/oLC8122), *RPT5* (oLC8092/oLC8093) and *PRE2* (oLC8162/oLC8163) are available in Table S3.

Protein extractions and western blotting

To monitor proteasome inhibition in different fungi, overnights of strains were subcultured to an OD_{600} of between 0.02 and 0.1 in 5 mL of YPD in the absence or presence of 130-260 µM bortezomib and incubated for 7 hours at 30°C with shaking. To monitor protein ubiquitination in strains with genetic depletion of the proteasome, cells were grown overnight in YPD in the absence of DOX or the presence of 0.05 µg/mL or 20 µg/mL DOX (as indicated) to repress target gene. Cells were then subcultured to an OD_{600} of between 0.1 and 0.2 in 10 mL of YPD with the same DOX conditions and grown for 4 hours at 30°C with shaking. Cells were harvested by centrifugation at 3000 rpm for 1 minute at 4°C and pellets were washed once with 1 mL of

distilled, cold water before being flash frozen in liquid nitrogen. The pellets were stored at -80°C. Cells were lysed by resuspending the pellet in 650 µL lysis buffer (0.42M NaOH and 1.9% β-mercaptoethanol) and placed on ice for 5 minutes. Proteins were precipitated by adding 150 µL of 50% trichloroacetic acid (TCA) and placed on ice for 5 minutes. Precipitates were collected at 14000 x *g* for 10 minutes at 4°C and pellets were washed once with 500 µL of ice-cold acetone. The supernatant was removed, and pellets were spun for 15 seconds on high to remove all remaining liquid. Pellets were resuspended in 150 µL loading buffer (40 mM Tris-HCl (pH 6.8), 5% (w/v) SDS, 100 mM NaEDTA, 8.3M Urea). Samples were heated at 45°C for 3 minutes and debris was pelleted by spinning for 1 minute at 12000 x *g*. Protein levels were determined by DC protein assay (Bio-Rad), diluted into 1 X sample buffer (one sixth volume of 6X sample buffer containing 40 mM Tris-HCl (pH 6.8), 5% (w/v) SDS, 100 mM NaEDTA, 8.3M Urea, 6% β-mercaptoethanol, and 0.06% bromophenol blue). 40 µg of protein per sample was separated by SDS-PAGE using precast 10-well Novex™ 4-12% Tris-Glycine Mini Gel (Invitrogen). 25 µg of protein per sample was separated by SDS-PAGE using precast 15-well Novex™ 4-12% Tris-Glycine Mini Gel (Invitrogen). Separated proteins were electrotransferred to polyvinylidene difluoride (PVDF) membrane (Bio-Rad) for 60 minutes at 300 mA. Following transfer, membranes were denatured in 20 mL Buffer A (10 mM Tris (pH 8.0), 6 M guanidium chloride, 100 mM NaH_2PO_4) for 30 minutes with gentle agitation. The membrane was washed extensively in phosphate-buffered saline (PBS) for 1 hour. Blots were blocked with 5% skim milk in PBS with 0.1% Tween 20 (PBS-T). Ubiquitin epitopes were detected using an anti-ubiquitin antibody (1:2,000, Santa Cruz Biotechnology P4D1, SC-8017, antibody ID, AB_628423) in blocking solution overnight at 4°C. Blots were washed with PBS-T and incubated with HRP-conjugated secondary antibody diluted 1:5,000 in block solution

for 40 minutes. Signals were detected using Clarity ECL HRP substrate kit as per the manufacturer's instructions (Bio-Rad). Membranes were washed once with distilled water and stained with 15 mL Ponceau (0.15% w/v Ponceau S (Sigma P3504), 40% methanol, 15% acetic acid) for 5 minutes. Membranes were washed 2-3 times with distilled water and imaged.

Dose response assays and spotting

Dose-response assays were performed in flat-bottom, 96-well microtiter plates (Sarstedt), as previously described (5). For target gene depletion in the *tetO* strains, cells were incubated overnight without DOX before being assayed for drug sensitivity in the presence of DOX, except for the cAMP-PKA pathway mutants. For these strains, cells were incubated overnight in 0.05 µg/mL DOX before being assayed for drug sensitivity. Assays were set up in a total volume of 0.1 mL/well with 2-fold or 5-fold dilutions of each drug, as indicated. Cell densities of overnight cultures were determined, and dilutions were prepared such that ~10^3 cells were inoculated into each well. Plates were incubated at 30°C for 48 hours before OD_{600} was measured using a spectrophotometer (Molecular Devices). Each strain was tested in technical and biological duplicates. Data were quantitatively displayed with color using Java TreeView 1.1.6r4 (http://jtreeview.sourceforge.net). To evaluate cidality, 5 µL of cells were transferred from the dose-response assays onto solid YPD medium containing no compound. Plates were photographed after 24 hours incubation at 30°C.

References

1. Roemer T, Jiang B, Davison J, Ketela T, Veillette K, Breton A, Tandia F, Linteau A, Sillaots S, Marta C, Martel N, Veronneau S, Lemieux S, Kauffman S, Becker J, Storms R, Boone C,

Bussey H. 2003. Large-scale essential gene identification in *Candida albicans* and applications to antifungal drug discovery. Mol Microbiol 50:167–181.

2. Xu D, Jiang B, Ketela T, Lemieux S, Veillette K, Martel N, Davison J, Sillaots S, Trosok S, Bachewich C, Bussey H, Youngman P, Roemer T. 2007. Genome-wide fitness test and mechanism-of-action studies of inhibitory compounds in *Candida albicans*. PLOS Pathog 3:e92.
3. Min K, Ichikawa Y, Woolford CA, Mitchell AP. 2016. *Candida albicans* gene deletion with a transient CRISPR-Cas9 system. mSphere 1:e00130-16.
4. Veri AO, Miao Z, Shapiro RS, Tebbji F, O'Meara TR, Kim SH, Colazo J, Tan K, Vyas VK, Whiteway M, Robbins N, Wong KH, Cowen LE. 2018. Tuning Hsf1 levels drives distinct fungal morphogenetic programs with depletion impairing Hsp90 function and overexpression expanding the target space. PLOS Genet 14:e1007270.
5. Singh SD, Robbins N, Zaas AK, Schell WA, Perfect JR, Cowen LE. 2009. Hsp90 governs echinocandin resistance in the pathogenic yeast *Candida albicans* via calcineurin. PLOS Pathog 5:e1000532.
