# Supplementary material for: The Proteasome Governs Fungal Morphogenesis via Functional Connections with Hsp90 and cAMP-Protein Kinase A Signaling
Source: mBio. 2020 Apr 21;11(2):e00290-20. doi: 10.1128/mBio.00290-20 (PMC7175089; doi:10.1128/mBio.00290-20)
Supplement: TABLE S1 [file mBio.00290-20-st001.pdf]

Table S1: Strains used in this study.

Strain Name	Alias	Genotype	Source
ScLC382	*S. cerevisiae* Sigma	*ura3Δ*	Kohler Lab
CkLC571	*Candida krusei* 1680	Clinical isolate	Heitman Lab
ClLC572	*C. lusitaniae* (ATCC® 42720™)	Clinical isolate	ATCC
CtLC575	*C. tropicalis* 1685	Clinical isolate	Heitman Lab
CdLC743	*C. dubliniensis* 36	Clinical isolate	(1)
CaLC5083	*C. auris* Ci6684	Clinical isolate	(2)
CpLC6144	*Candida parapsilosis* (ATCC® 22019™)	Clinical isolate	ATCC
CaLC239	SN95	*arg/arg4 his1/his1 URA3/ura3::imm*^{434} *IRO1/iro1::imm*^{434}	(3)
CaLC555	CAI4 *cyr1Δ/cyr1Δ*	*ura3::imm*^{434}*/ura3::imm*^{434} *cyr1::hisG-URA3-hisG/cyr1::hisG*	(4)
CaLC563	CAI4 *efg1Δ/efg1Δ*	*ura3::imm*^{434}*/ura3::imm*^{434} *efg1::hisG/efg1::hisG-URA3-hisG*	(5)
CaLC564	CAI4 *ras1Δ/ras1Δ*	*ura3::imm*^{434}*/ura3::imm*^{434} *ras1::hisG/ras1::hph*	(6)
CaLC914	CAI4 *cph1Δ/cph1Δ*	*ura3::imm*^{434}*/ura3::imm*^{434} *cph1::hisG/cph1::hisG-URA3-hisG*	(7)
CaLC922	CAI4 *HSP70p-lacZ*	*ura3::imm*^{434}*/ura3::imm*^{434} *HSP70/HSP70p-lacZ::URA3*	(8)
CaLC1566	SN152 *ume6Δ/ume6Δ*	*ume6::C. maltosa LEU2/ume6::C. dubliniensis HIS1 arg4::ARG4/arg4 leu2/leu2 his1/his1 ura3::imm*^{434}*/URA3 iro1::imm*^{434}*/IRO1*	(9)
CaLC2445	CAI4 *cek1Δ/cek1Δ*	*ura3/ura3 cek1::hisG-URA3-hisG/cek1::hisG*	(10)
CaLC2560/ CaLC2561	SN95 *tec1Δ/tec1Δ*	*arg4/arg4 his1/his1 URA3/ura3::imm*^{434} *IRO1/iro1::imm*^{434} *tec1::FRT/tec1::FRT*	(11)
CaLC2676	SN95 *bcr1Δ/bcr1Δ*	*arg4 /arg4 his1/his1 URA3/ura3::imm*^{434} *IRO1/iro1::imm*^{434} *bcr1::FRT/bcr1::FRT*	(11)

CaLC2737	SN152 *brg1Δ/brg1Δ*	*arg4/arg4 leu2/leu2 his1/his1 URA3/ura3::imm^{434} IRO1/iro1::imm^{434} brg1::C. albicans HIS1/brg1::C. albicans LEU2*	(12)
CaLC2740	SN152 (Homann library WT)	*arg4/arg4 leu2/leu2::C. albicans LEU2 his1/his1::C. albicans HIS1 URA3/ura3::imm^{434} IRO1/iro1::imm^{434}*	(12)
CaLC2742	CAF2-1	*ura3::imm^{434}/URA3*	(13)
CaLC2897	SN95 *flo8Δ/flo8Δ*	*arg4/arg4 his1/his1 URA3/ura3::imm^{434} IRO1/iro1::imm^{434} flo8::FRT/flo8::FRT*	(14)
CaLC3067	SN95 Hsp104-GFP	*arg/arg4 his1/his1 URA3/ura3::imm^{434} IRO1/iro1::imm^{434} HSP104/HSP104-GFP*	(15)
CaLC3431	SN152 *ace2Δ/ace2Δ*	*arg4/arg4 leu2/leu2 his1/his1 URA3/ura3::imm^{434} IRO1/iro1::imm^{434} ace2::C. albicans HIS1/ace2::C. albicans LEU2*	(12)
CaLC4506	SN95 Nop1-GFP	*arg4 /arg4 his1 /his1 URA3/ura3::imm^{434} IRO1/iro1::imm^{434} NOP1-GFP-HIS/NOP1*	(14)
CaLC5039	SN152 *tetO-HSF1/tetO-HSF1 ume6Δ/ume6Δ* (preflip)	*ume6::C. maltosa LEU2/ume6::C. dubliniensis HIS1 arg4::ARG4/arg4 leu2/leu2 his1/his1 ura3 ::imm^{434}/URA3 iro1 ::imm^{434}/IRO1 NEUT5L-FRT-ENO1p-Cas9-NAT-snR52p-HSF1p sgRNA-FRT/NEUT5L TAR-tetO-HSF1/TAR-tetO-HSF1*	(16)
CaLC5084	SN152HLA (WT)	*arg4::hisG/ARG4, his1::hisG/HIS1, leu2::hisG/LEU2 ura3::λimm434::URA3-IRO1/ura3:: λimm434*	(17)
CaLC5088	SN152 *tpk1Δ/tpk1Δ tpk2Δ/tpk2Δ*	*ura3::λimm434::URA3-IRO1/ura3:: λimm434, tpk2::HIS1/ tpk2::ARG4, tpk1::LEU2/ tpk1::FRT*	(17)
CaLC5275	SN250 *tetO-HSF1/tetO-HSF1* (preflip)	*arg4/arg4 his1/his1 leu2::C.dubliniensis HIS1/leu2::C.maltosa LEU2 URA3/ura3::imm^{434} IRO1/iro1::imm^{434}*	(17)

		NEUT5L-FRT-ENO1p-Cas9-NAT-snR52p-HSF1p sgRNA-FRT/NEUT5L TAR-tetO-HSF1/TAR-tetO-HSF1	
CaLC5277	SN152 *tetO-HSF1/tetO-HSF1 brg1Δ/brg1Δ* (preflip)	*arg4/arg4 leu2/leu2 his1/his1 URA3/ura3::imm^{434} IRO1/iro1 ::imm^{434} brg1::C. albicans HIS1/ brg1::C. albicans LEU2 NEUT5L-FRT-ENO1p-Cas9-NAT-snR52p-HSF1p sgRNA-FRT/NEUT5L TAR-tetO-HSF1/TAR-tetO-HSF1*	(17)
CaLC6152	CAF2-1 *tetO-RPT5/tetO-RPT5*	*ura3::imm^{434}/URA3 TAR-FRT::tetO-RPT5/ TAR-FRT::tetO-RPT5*	This study
CaLC6153	CAI4 *tetO-RPT5/tetO-RPT5 efg1Δ/efg1Δ*	*ura3::imm^{434}/ura3::imm^{434} efg1::hisG/efg1::hisG-URA3-hisG TAR-FRT::tetO-RPT5/ TAR-FRT::tetO-RPT5*	This study
CaLC6196	CAI4 *ras1Δ/ras1Δ*	*ura3::imm^{434}/ura3::imm^{434} ras1::hisG/ras1::hph TAR-FRT::tetO-RPT5/ TAR-FRT::tetO-RPT5*	This study
CaLC6227	CAI4 *cyr1Δ/cyr1Δ*	*ura3::imm^{434}/ura3::imm^{434} cyr1::hisG-URA3-hisG/cyr1::hisG TAR-FRT::tetO-RPT5/ TAR-FRT::tetO-RPT5*	This study
CaLC6106	GRACE parent strain (CaSS1)	*ura3::imm^{434}/ura3::imm^{434} his3::hisG/his3::hisG leu2::tetR-GAL4AD-URA/LEU2*	(18)
GRACE strain	*tetO-PRE1/pre1Δ*	As GRACE parent *SAT1::tetO-PRE1/ pre1::HIS3*	(18)
GRACE strain	*tetO-PRE2/pre2Δ*	As GRACE parent *SAT1::tetO-PRE2/ pre2::HIS3*	(18)
GELC0586	*tetO-PRE3/pre3Δ*	As GRACE parent *SAT1::tetO-PRE3/ pre3::HIS3*	This Study
GELC0394	*tetO-PRE4/pre4Δ*	As GRACE parent *SAT1::tetO-PRE4/ pre4::HIS3*	This Study
GRACE strain	*tetO-PRE5/pre5Δ*	As GRACE parent *SAT1::tetO-PRE5/ pre5::HIS3*	(18)
GRACE strain	*tetO-PRE6/pre6Δ*	As GRACE parent *SAT1::tetO-PRE6/ pre6::HIS3*	(18)
GRACE strain	*tetO-PRE7/pre7Δ*	As GRACE parent *SAT1::tetO-PRE7/ pre7::HIS3*	(18)
GRACE strain	*tetO-PRE8/pre8Δ*	As GRACE parent *SAT1::tetO-PRE8/ pre8::HIS3*	(18)

GELC0910	*tetO-PRE9/pre9Δ*	As GRACE parent *SAT1::tetO-PRE9/ pre9Δ::HIS3*	This Study
GELC0118	*tetO-PRE10/pre10Δ*	As GRACE parent *SAT1::tetO-PRE10/ pre10Δ::HIS3*	This Study
GRACE strain	*tetO-PUP1/pup1Δ*	As GRACE parent *SAT1::tetO-PUP1/ pup1::HIS3*	(18)
GRACE strain	*tetO-PUP2/pup2Δ*	As GRACE parent *SAT1::tetO-PUP2/ pup2::HIS3*	(18)
GELC0583	*tetO-PUP3/pup3Δ*	As GRACE parent *SAT1::tetO-PUP3/ pup3::HIS3*	This study
GRACE strain	*tetO-SCL1/scl1Δ*	As GRACE parent *SAT1::tetO-SCL1/ scl1::HIS3*	(18)
GRACE strain	*tetO-RPN3/rpn3Δ*	As GRACE parent *SAT1::tetO-RPN3/ rpn3::HIS3*	(18)
GRACE strain	*tetO-RPN5/rpn5Δ*	As GRACE parent *SAT1::tetO-RPN5/ rpn5::HIS3*	(18)
GRACE strain	*tetO-RPN6/rpn6Δ*	As GRACE parent *SAT1::tetO-RPN6/ rpn6::HIS3*	(18)
GRACE strain	*tetO-RPN7/rpn7Δ*	As GRACE parent *SAT1::tetO-RPN7/ rpn7::HIS3*	(18)
GRACE strain	*tetO-RPN8/rpn8Δ*	As GRACE parent *SAT1::tetO-RPN8/ rpn8::HIS3*	(18)
GELC0058	*tetO-RPN9/rpn9Δ*	As GRACE parent *SAT1::tetO-RPN9/ rpn9::HIS3*	This Study
GELC0588	*tetO-SEM1/sem1Δ*	As GRACE parent *SAT1::tetO-SEM1/ SEM1::HIS3*	This Study
GRACE strain	*tetO-RPN11/rpn11Δ*	As GRACE parent *SAT1::tetO-RPN11/ rpn11::HIS3*	(18)
GRACE strain	*tetO-RPN12/rpn12Δ*	As GRACE parent *SAT1::tetO-RPN12/ rpn12::HIS3*	(18)
GRACE strain	*tetO-RPT1/rpt1Δ*	As GRACE parent *SAT1::tetO-RPT1/ rpt1::HIS3*	(18)
GELC0395	*tetO-RPT2/rpt2Δ*	As GRACE parent *SAT1::tetO-RPT2/ rpt2::HIS3*	This Study
GRACE strain	*tetO-RPT3/rpt3Δ*	As GRACE parent *SAT1::tetO-PR26/ pr26::HIS3*	(18)
GRACE strain	*tetO-RPT4/rpt4Δ*	As GRACE parent *SAT1::tetO-RPT4/ rpt4::HIS3*	(18)
GRACE strain	*tetO-RPT5/rpt5Δ*	As GRACE parent *SAT1::tetO-RPT5/ rpt5::HIS3*	(18)
GRACE strain	*tetO-RPT6/rpt6Δ*	As GRACE parent *SAT1::tetO-RPT6/ rpt6::HIS3*	(18)

GRACE strain	*tetO-RPN1/rpn1Δ*	As GRACE parent *SAT1::tetO-RPN1/ rpn1::HIS3*	(18)
GRACE strain	*tetO-RPN2/rpn2Δ*	As GRACE parent *SAT1::tetO-RPN2/ rpn2::HIS3*	(18)
GELC0260	*tetO-RPN10/rpn10Δ*	As GRACE parent *SAT1::tetO-RPN10/ rpn10::HIS3*	This study
GRACE strain	*tetO-RPN13/rpn13Δ*	As GRACE parent *SAT1::tetO-RPN13/ rpn13::HIS3*	(18)
HELC0260	*RPN10/rpn10Δ*	As GRACE parent *RPN10/rpn10::HIS3*	This study
HELC0583	*PUP3/pup3Δ*	As GRACE parent *PUP3/pup3::HIS3*	This study
HELC0586	*PRE3/pre3Δ*	As GRACE parent *PRE3/pre3::HIS3*	This Study
HELC0588	*SEM1/sem1Δ*	As GRACE parent *SEM1/SEM1::HIS3*	This Study
HELC0910	*PRE9/pre9Δ*	As GRACE parent *PRE9/pre9Δ::HIS3*	This Study

References:

1. Sullivan DJ, Westerneng TJ, Haynes KA, Bennett DE, Coleman DC. 1995. *Candida dubliniensis* sp. nov.: phenotypic and molecular characterization of a novel species associated with oral candidosis in HIV-infected individuals. Microbiol Read Engl 141 (Pt 7):1507–1521.
2. Chatterjee S, Alampalli SV, Nageshan RK, Chettiar ST, Joshi S, Tatu US. 2015. Draft genome of a commonly misdiagnosed multidrug resistant pathogen *Candida auris*. BMC Genomics 16:686.
3. Noble SM, Johnson AD. 2005. Strains and strategies for large-scale gene deletion studies of the diploid human fungal pathogen *Candida albicans*. Eukaryot Cell 4:298–309.
4. Jain P, Akula I, Edlind T. 2003. Cyclic AMP signaling pathway modulates susceptibility of *Candida* species and *Saccharomyces cerevisiae* to antifungal azoles and other sterol biosynthesis inhibitors. Antimicrob Agents Chemother 47:3195–3201.
5. Braun BR, Johnson AD. 2000. *TUP1*, *CPH1* and *EFG1* make independent contributions to filamentation in *Candida albicans*. Genetics 155:57–67.
6. Feng Q, Summers E, Guo B, Fink G. 1999. Ras signaling is required for serum-induced hyphal differentiation in *Candida albicans*. J Bacteriol 181:6339–6346.
7. Liu H, Kohler J, Fink GR. 1994. Suppression of hyphal formation in *Candida albicans* by mutation of a STE12 homolog. Science 266:1723–1726.
8. Shapiro RS, Uppuluri P, Zaas AK, Collins C, Senn H, Perfect JR, Heitman J, Cowen LE. 2009. Hsp90 orchestrates temperature-dependent *Candida albicans* morphogenesis via Ras1-PKA Signaling. Curr Biol 19:621–629.
9. Banerjee M, Thompson DS, Lazzell A, Carlisle PL, Pierce C, Monteagudo C, López-Ribot JL, Kadosh D. 2008. *UME6*, a novel filament-specific regulator of *Candida albicans* hyphal extension and virulence. Mol Biol Cell 19:1354–1365.

10. Csank C, Schröppel K, Leberer E, Harcus D, Mohamed O, Meloche S, Thomas DY, Whiteway M. 1998. Roles of the *Candida albicans* mitogen-activated protein kinase homolog, Cek1p, in hyphal development and systemic candidiasis. Infect Immun 66:2713–2721.

11. Diezmann S, Leach MD, Cowen LE. 2015. Functional divergence of Hsp90 genetic interactions in biofilm and planktonic cellular states. PLOS ONE 10:e0137947.

12. Homann OR, Dea J, Noble SM, Johnson AD. 2009. A phenotypic profile of the *Candida albicans* regulatory network. PLoS Genet 5:e1000783.

13. Fonzi WA, Irwin MY. 1993. Isogenic strain construction and gene mapping in *Candida albicans*. Genetics 134:717–728.

14. Xie JL, O'Meara TR, Polvi EJ, Robbins N, Cowen LE. 2017. Staurosporine induces filamentation in the human fungal pathogen *Candida albicans* via signaling through Cyr1 and protein kinase A. mSphere 2:e00056-17.

15. Leach MD, Kim T, DiGregorio SE, Collins C, Zhang Z, Duennwald ML, Cowen LE. 2017. *Candida albicans* is resistant to polyglutamine aggregation and toxicity. G3 Genes Genomes Genet 7:95–108.

16. Veri AO, Miao Z, Shapiro RS, Tebbji F, O'Meara TR, Kim SH, Colazo J, Tan K, Vyas VK, Whiteway M, Robbins N, Wong KH, Cowen LE. 2018. Tuning Hsf1 levels drives distinct fungal morphogenetic programs with depletion impairing Hsp90 function and overexpression expanding the target space. PLOS Genet 14:e1007270.

17. Cao C, Wu M, Bing J, Tao L, Ding X, Liu X, Huang G. 2017. Global regulatory roles of the cAMP/PKA pathway revealed by phenotypic, transcriptomic and phosphoproteomic analyses in a null mutant of the PKA catalytic subunit in *Candida albicans*. Mol Microbiol 105:46–64.

18. Roemer T, Jiang B, Davison J, Ketela T, Veillette K, Breton A, Tandia F, Linteau A, Sillaots S, Marta C, Martel N, Veronneau S, Lemieux S, Kauffman S, Becker J, Storms R, Boone C, Bussey H. 2003. Large-scale essential gene identification in *Candida albicans* and applications to antifungal drug discovery. Mol Microbiol 50:167–181.
