# Supplementary material for: The Proteasome Governs Fungal Morphogenesis via Functional Connections with Hsp90 and cAMP-Protein Kinase A Signaling
Source: mBio. 2020 Apr 21;11(2):e00290-20. doi: 10.1128/mBio.00290-20 (PMC7175089; doi:10.1128/mBio.00290-20)
Supplement: TABLE S2 [file mBio.00290-20-st002.pdf]

Table S2: Plasmids used in this study.

Plasmid	Description	Source
pLC49	p863 (for gene disruption with *FLP-NAT*)	(1)
pLC605	*CaTAR-FLP-NAT-tetO*	(2)
pLC763	*Ca-FLP-NAT-tetO*	This study
pLC963	pV1393-1 (CaCas9/sgRNA entry expression vector, contains NatR gene, targeting *NEUT5L*)	(2)
pLC1237	pUC19 Vector	NEB (3)
pLC1251	*Ca-HIS3* (for gene disruption)	This study

References:

1. Shen J, Guo W, Köhler JR. 2005. *CaNAT1*, a heterologous dominant selectable marker for transformation of *Candida albicans* and other pathogenic *Candida* species. Infect Immun 73:1239–1242.

2. Veri AO, Miao Z, Shapiro RS, Tebbji F, O'Meara TR, Kim SH, Colazo J, Tan K, Vyas VK, Whiteway M, Robbins N, Wong KH, Cowen LE. 2018. Tuning Hsf1 levels drives distinct fungal morphogenetic programs with depletion impairing Hsp90 function and overexpression expanding the target space. PLOS Genet 14:e1007270.

3. Yanisch-Perron C, Vieira J, Messing J. 1985. Improved M13 phage cloning vectors and host strains: nucleotide sequences of the M13mpl8 and pUC19 vectors. Gene 33:103–119.
