# Supplementary material for: The Proteasome Governs Fungal Morphogenesis via Functional Connections with Hsp90 and cAMP-Protein Kinase A Signaling
Source: mBio. 2020 Apr 21;11(2):e00290-20. doi: 10.1128/mBio.00290-20 (PMC7175089; doi:10.1128/mBio.00290-20)
Supplement: TABLE S3 [file mBio.00290-20-st003.pdf]

Table S3: Oligonucleotides used in this study.

Name	Description	Sequence (5' -> 3')
GRACE and HET strain construction		
oLC118.1	*PRE10* Primer1	ACAAACTTACTCTCTTCTTCTTTTGTAATCATTACAACAATTCTTATATAACTAATATTTTATCTATATCACTGGATGGCGGCGTTAGTATC
oLC118.2	*PRE10* Primer2	TGAAGTCTATAAATCAGTTAGTATACATGAGCAATGCATTGTGTTTAGTTGTAACAAATTGACATACCATCGACTATTTATATTTGTATG
oLC118.3	*PRE10* Primer3	GCCATTTTAACTGTACTGCCAT
oLC118.4	*PRE10* Primer4	TTTTCAACGGCTTTCATGGCAT
oLC394.1	*PRE4* Primer1	ATTGCATTTAAGTTTTTATATTTTGTATAACTTTTTTTTTGAAAAACAAGAAGAAAACAATCACGAAAAACTGGATGGCGGCGTTAGTATC
oLC394.2	*PRE4* Primer2	CAATTTTGTAATTGTATGGACCATAGGTGTCATTGGATGGTCTACCCCAACTGTATGGATCGTGATTCATCGACTATTTATATTTGTATG
oLC394.3	*PRE4* Primer3	GGTGTAAGCTTTACAGTGGTTT
oLC394.4	*PRE4* Primer4	ACCAACAACAGTCTCCTTACCT
oLC395.1	*RPT2* Primer1	TCTTATTTCTTGGTGGCGAGTTTTCCAAATCAAACATTATTTGTATATCCCATACTCCAAGCAAGCAATCACTGGATGGCGGCGTTAGTATC
oLC395.2	*RPT2* Primer2	CCTTTTTCTTATCATCTTTCTTTTTGTTGAGATTATCGCCACCTGGTATTCCTGAAGGACCTTGACCCATCGACTATTTATATTTGTATG
oLC395.3	*RPT2* Primer3	ATGCTTGTCAAGAAACACATCC
oLC395.4	*RPT2* Primer4	TGCTTCTGTAGGTTGGAATGCT
oLC58.1	*RPN9* Primer1	AACCAACCAACCAAGACAACTTATCTTCTTCTTATAGACCACCCTTAAGATCAACAACAATATGTCGCCAACTGGATGGCGGCGTTAGTATC
oLC58.2	*RPN9* Primer2	TTAATTCAGAATTATCTGATTTCAGCCTAATTGTTGCTAAAACTGTAGAAACTTCAGTATCTATATCCATCGACTATTTATATTTGTATG
oLC58.3	*RPN9* Primer3	TTATTTCAAGTGCCTGCTTTGA
oLC58.4	*RPN9* Primer4	CAACATCATCAGTAACTCTATAATTATGAC
oLC260.1	*RPN10* Primer1	TTCAACAGTGTTGATTTTCAATTCTTTACCCTATCCCTAAAACGTACAGTATTATATTAAAGAAACAAAAACTGGATGGCGGCGTTAGTATC
oLC260.2	*RPN10* Primer2	ATGTCAAAAAGTCACCATTCCTCATATACTCAGAGTTGTCAATGGCAATCATAGTAGCCTCAAGAACCATCGACTATTTATATTTGTATG

oLC260.3	*RPN10* Primer3	TATATGAGCAGTTCTTGGACGT
oLC260.4	*RPN10* Primer4	CAATGCTGCTACTTGGATTCCG
oLC260.5	*RPN10* Primer5	ACCCTATCCCTAAAACGTACAGTATTATATTAAAGAAACAAAAGATGTCCACGAGGTCTCTAGACCTTGCACATGCTTGCGCGTACGCTGCAGGTCGAC
oLC260.6	*RPN10* Primer6	CGAATATAGTATTATTTTTATTATGCTTCATATTCTATACAGCCGGTGTCGGTCTCGTAGAGACTTCGATCATGCTCTTGATCGATGAATTCGAGCTCG
oLC583.1	*PUP3* Primer1	AGTGGACCCAATTGAATAGAATATCTCAATAGATTATAAATTACTATCCTCATTCTCAATTTAACAGATCACTGGATGGCGGCGTTAGTATC
oLC583.2	*PUP3* Primer2	CTATTGCTATACAGTCTTTACCAATCATAGCTACTGCTGCACCTCCGTTAATTGAAAATGGATCCGACATCGACTATTTATATTTGTATG
oLC583.3	*PUP3* Primer3	GAGATTTTGTACAAGTATCTTCATTG
oLC583.4	*PUP3* Primer4	ACCAAATTTGCCAATGTTGATG
oLC583.5	*PUP3* Primer5	AGATTATAAATTACTATCCTCATTCTCAATTTAACAGATCATGGATGTCCACGAGGTCTCTTCAAATTCATTAGTTTAGAACGTACGCTGCAGGTCGAC
oLC583.6	*PUP3* Primer6	TCAAATCATGTATTATCTGCTAACTTTATAACATATAAATCAACGGTGTCGGTCTCGTAGAACCGCTGTACGGTGATTGCATCGATGAATTCGAGCTCG
oLC586.1	*PRE3* Primer1	AAAAAAAAACTAGTGGCAAACCACCTTAATCAACAATTTCCTAACATCATCAAACATAGCATAATCAACAACTGGATGGCGGCGTTAGTATC
oLC586.2	*PRE3* Primer2	CCATAATTGAAGTACCTAAATTCACTTCCCCCTTTTTCAAATGATTGATGTCTACTGAAATACCGTTCATCGACTATTTATATTTGTATG
oLC586.3	*PRE3* Primer3	TGTTGTTGTTGTTGTTGAATGG
oLC586.4	*PRE3* Primer4	CCAGGAGGCAATTGTGATGAAT
oLC586.5	*PRE3* Primer5	CAACAATTTCCTAACATCATCAAACATAGCATAATCAACAATGGATGTCCACGAGGTCTCTCGTGTGCGACGTCTCACTGGCGTACGCTGCAGGTCGAC
oLC586.6	*PRE3* Primer6	CAGATTATCTTATTCTATCTACCGAATATAAAAGCATTTTTATCGGTGTCGGTCTCGTAGTTGTCCCACCGCCTACGTGAATCGATGAATTCGAGCTCG
oLC588.1	*SEM1* Primer1	GAACGAGGAAAAAAAAAAATGTCATGAGTGGCAACCACTACTTAGTCAACATCAAATCAATAGTACCATAACTGGATGGCGGCGTTAGTATC
oLC588.2	SEM1 Primer2	TGAGACCTGATGTATGTTTCAGAGGGATTGTAGTAGATGGTTTAGAATCTAGTTTGGTGGCGTCTGACATCGACTATTTATATTTGTATG
oLC588.3	*SEM1* Primer3	ACAATATAGCCCAGCCTGATTT

oLC588.4	*SEM1* Primer4	AGACACTTCATCTTGACCATCA
oLC588.5	*SEM1* Primer5	GCAACCACTACTTAGTCAACATCAAATCAATAGTACCATAATGGATGTCCACGAGGTCTCTACTGGAGCTGTCTACATCCTCGTACGCTGCAGGTCGAC
oLC588.6	*SEM1* Primer6	CCTTTTCCTGTTAAATAATCAACTAAGAAAGCATCTAAACTATCGGTGTCGGTCTCGTAGCGCTGTCGCGATGGACTTGTATCGATGAATTCGAGCTCG
oLC910.1	*PRE9* Primer1	TTTAAGAACTGATACTGTTGAAACTAAAGTACTACAAAACTATTATCAAAGCTAAACAAGACCATATACCACTGGATGGCGGCGTTAGTATC
oLC910.2	*PRE9* Primer2	CATATTCCACTTGGTATAATCTACCTTCTGGTGAAAAAATAGTGGTTCTTGAATCGTATCTTCTTGACATCGACTATTTATATTTGTATG
oLC910.3	*PRE9* Primer3	AAATTGGTGATCTGCGACATTT
oLC910.4	*PRE9* Primer4	CTGTTGGGCTTGAATTCTTGCA
oLC910.5	*PRE9* Primer5	AGTACTACAAAACTATTATCAAAGCTAAACAAGACCATATACCGATGTCCACGAGGTCTCTCACCTATCAGGAGACGTGAGCGTACGCTGCAGGTCGAC
oLC910.6	*PRE9* Primer6	ACACTAAATCTAAACACTGGTAGTCCTATAAACATCAGTATATCGGTGTCGGTCTCGTAGGGCCTATTGTCAGTGCCCTTATCGATGAATTCGAGCTCG
Primers for *tetO-RPT5*		
oLC534	CaTAR-797-R	GATGGAGATAGTTTACGG
oLC4714	tetOp+488F	TCGTTTCTGATGGGCTTTTC
oLC5978	pLC963-SNR52-F	GACTGTCAAGGAGGGTATTC
oLC5979	pLC963-SNR52-N-F	CCGCAAGTGATTAGACTTAG
oLC5980	pLC963-sgRNA-R	GAATACCACTTGTTTACCGG
oLC5981	pLC963-sgRNA-N-R	GGTGGCGGCAAAACTAATTC
oLC6924	*CaCas9*/for	ATCTCATTAGATTTGGAACTTGTGGGTT
oLC6925	*CaCas9*/rev	TTCGAGCGTCCCAAAACCTTCT
oLC7838	*CaRPT5*-pLC605-R	TCAAAATTTCTTGATCTATAACATTTTCTTCTTGTTGCTGATTGTTTTCTAAATCTTCTAAGGTAACCATCGACTATTTATATTTGTATG
oLC7839	*CaRPT5*-pLC605-F	GGGAGAAAAAAAAAATGCTAGGGAAAGACTCGACCTCTCCCAATTACAGAATACTACAACTGTATCAACAGGAAACAGCTATGACCATG
oLC7840	*CaRPT5*-320-F-AB	AATAAGCGGCGGATTTGG
oLC7841	*CaRPT5*+304R-AB	GCTCTAGCTGCGTCGATAT
oLC7842	*CaRPT5*_sgRNA_F	TACTACAACTGTATCAACAAGTTTTAGAGCTAGAAATAGCAAGTTAAA
oLC7843	*CaRPT5*_sgRNA_R	TTGTTGATACAGTTGTAGTACAAATTAAAAATAGTTTACGCAAGTC
RT PCR Primers		

oLC8159	*CaRPN9*+AB+808F	CGGCTGAAACTAATTCTAAT
oLC8161	*CaRPN9*+AB+981R	CCTCAATTAGAATTAGCTCC
oLC2285	*CaACT1*AB+855-F	GACCTTGAGATACCCAATTG
oLC2286	*CaACT1*AB+1076-R	CAGCTTGAATGGAAACGTAG
oLC752	*GPD1*A+570-F	AGTATGTGGAGCTTTACTGGGA
oLC753	*GPD1*AB+766-R	CAGAAACACCAGCAACATCTTC
oLC8121	*CaRPN13*-AB+704R	GTGACATCAAATACCCTGCTC
oLC8122	*CaRPN13*-AB+589F	CTTCCTCCATCAATTGCTTC
oLC8092	*CaRPT5*_AB+233F	TGGTGGGAAATGTTGTGGAG
oLC8093	*CaRPT5*_AB+373R	GTCAACTAACCCTATCATTGG
oLC8485	*CaRPN10*_AB+129F	AACCCTGAAAATACAGTCGG
oLC8486	*CaRPN10*_AB+311R	TGATTCTTTGCTGTTGGACC
oLC8488	*CaPRE9*_AB+322F	CGACGAAGAGATTCCTTGTG
oLC8489	*CaPRE9*_AB+477R	CACCAATACTAGTTGCCTTC
oLC8491	*CaSEM1*_AB+049F	ACCATCTACTACAATCCCTC
oLC8492	*CaSEM1*_AB+191R	ATCATCCCAATCTTCTTCCC
Additional primers		
oLC2535	*TetOp*-F	GTTTGGTTCAGCACCTTGTCG
oLC6853	*SAT1_R*	CGCAGAAAGTAATATCATGC
oLC6701	*CaHIS3*prom_UPtag_R10	CCACCATCTAAATTAAGGGC
oLC7860	BamHI_*HIS3*_F	CGCGGATCCCGTACGCTGCAGGTCGAC
oLC7861	*HIS3*_BamHI_R	CGCGGATCCATCGATGAATTCGAGCTCG
